# Structural and Mechanistic Basis for the Inhibition of *Escherichia coli* RNA Polymerase by T7 Gp2

**DOI:** 10.1016/j.molcel.2012.06.013

**Published:** 2012-09-14

**Authors:** Ellen James, Minhao Liu, Carol Sheppard, Vladimir Mekler, Beatriz Cámara, Bing Liu, Pete Simpson, Ernesto Cota, Konstantin Severinov, Steve Matthews, Sivaramesh Wigneshweraraj

**Affiliations:** 1 MRC Centre for Molecular Bacteriology and Infection, Imperial College London, London SW7 2AZ, UK; 2 Waksman Institute for Microbiology and Department of Molecular Biology and Biochemistry, Rutgers, The State University of New Jersey, Piscataway, NJ 08854, USA; 3 Laboratorio de Microbiología Molecular y Biotecnología Ambiental, Departamento de Química, Universidad Técnica Federico Santa María, Valparaíso, Chile; 4 Institute of Molecular Genetics, Russian Academy of Science, Moscow 123182, Russia; 5 Institute of Gene Biology, Russian Academy of Science, Moscow 11991, Russia

## Abstract

The T7 phage-encoded small protein Gp2 is a non-DNA-binding transcription factor that interacts with the jaw domain of the *Escherichia coli* (*Ec*) RNA polymerase (RNAp) β′ subunit and inhibits transcriptionally proficient promoter-complex (RPo) formation. Here, we describe the high-resolution solution structure of the Gp2-*Ec* β′ jaw domain complex and show that Gp2 and DNA compete for binding to the β′ jaw domain. We reveal that efficient inhibition of RPo formation by Gp2 requires the amino-terminal σ^70^ domain region 1.1 (R1.1), and that Gp2 antagonizes the obligatory movement of R1.1 during RPo formation. We demonstrate that Gp2 inhibits RPo formation not just by steric occlusion of the RNAp-DNA interaction but also through long-range antagonistic effects on RNAp-promoter interactions around the RNAp active center that likely occur due to repositioning of R1.1 by Gp2. The inhibition of *Ec* RNAp by Gp2 thus defines a previously uncharacterized mechanism by which bacterial transcription is regulated by a viral factor.

## Highlights

► The structure of the T7 transcription factor Gp2-*E. coli* RNAp β′jaw domain complex is described ► Gp2 and DNA compete for overlapping interaction surface on the β′ jaw domain ► Gp2 requires σ^70^ region 1.1 to inhibit the *E. coli* RNAp efficiently ► Gp2 inhibits the obligatory displacement of σ^70^ region 1.1

## Introduction

**Figure 1:**
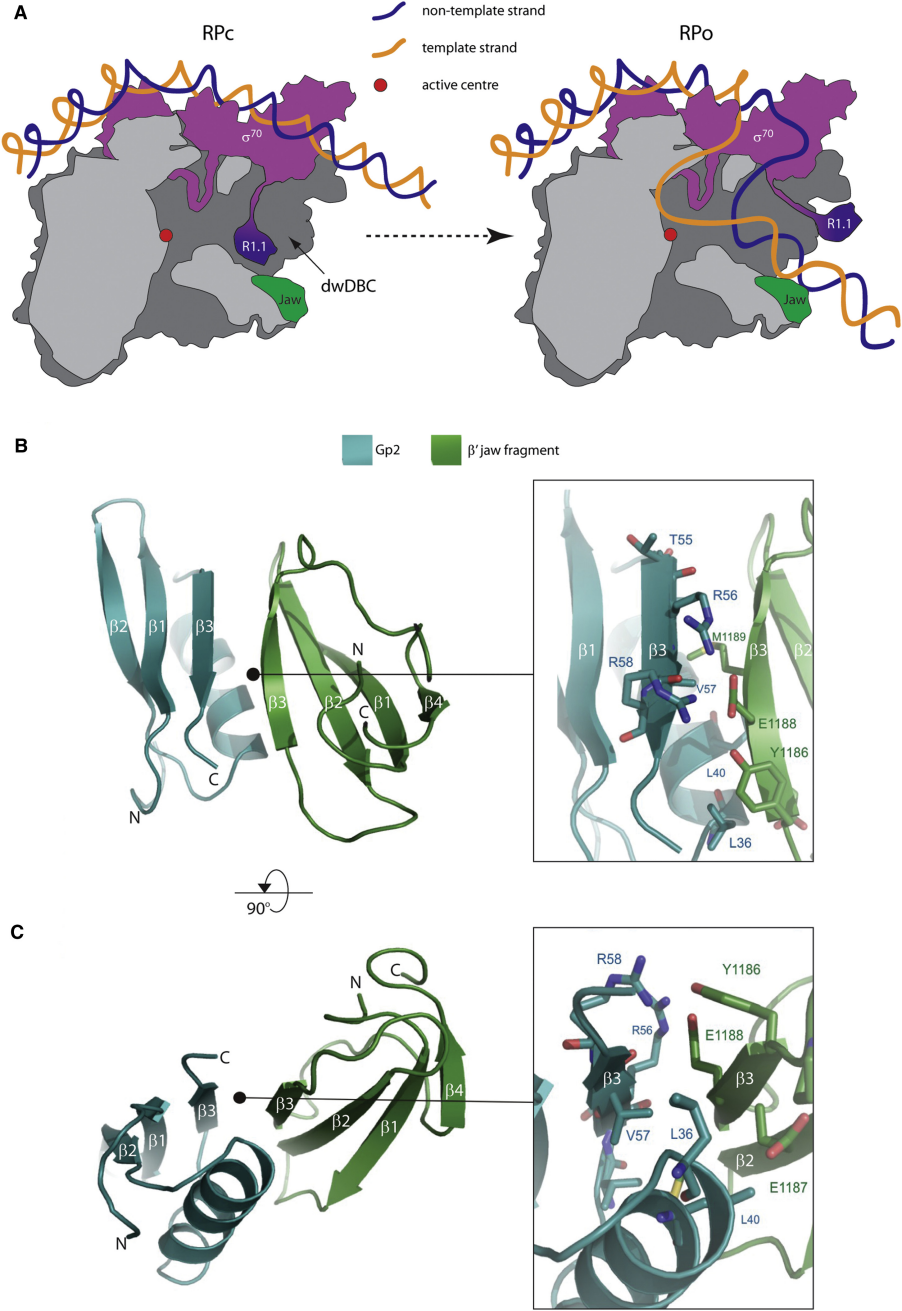
RPc and RPo Formation and the Structure of the Gp2-β′ Jaw Fragment Complex (A) Cartoon depiction of RPc and RPo formation at σ^70^-dependent bacterial promoters (the inspiration for the cartoon was taken from Murakami and Darst [2003]). (B) Ribbon representation of the Gp2-β′ jaw fragment complex. (C) The same as (B) but rotated by 90° along the horizontal plane. In (B) and (C), the interface region is enlarged in the insets, and the residues located at the interaction interface are shown as sticks and labeled correspondingly. See also Figure S1.

Transcription of DNA is a major focal point of regulation of gene expression in all organisms. In bacteria, transcription is catalyzed by a multisubunit RNA polymerase (RNAp) with subunit composition α_2_ββ′ωσ (abbreviated as Eσ). The catalytic determinants of the bacterial RNAp are contained in the largest and second-largest subunits (β′ and β, respectively). The overall architecture of RNAp is reminiscent of a crab claw (Murakami et al., 2002b). The two pincers of the claw form a positively charged DNA binding channel (DBC). The active center where RNA synthesis occurs is located deep within the DBC (Figure 1A). A number of flexible domains from the β′ and β subunits (β′ jaw, β′ insertion 6, β′ downstream clamp, and β downstream and upstream lobe domains) surround the DBC and contribute to stable binding of DNA (Opalka et al., 2010).

A dissociable RNAp binding factor called the sigma (σ) subunit confers promoter specificity upon the RNAp by recognizing the −35 and −10 (with respect to the transcription start site at +1, hereafter called the +1 site) elements present in most bacterial promoters. Of the seven σ factors encoded by the *Escherichia coli* (*Ec*) genome, σ^70^ is responsible for transcription of housekeeping genes and is therefore a major σ factor essential for growth of the cell (Haugen et al., 2008). The six remaining “alternative” σ factors contribute to transcription of genes under specific stress conditions, growth transitions, and/or morphological changes (Gruber and Gross, 2003). Other bacteria also have one σ^70^-like major σ factor that is responsible for transcription of housekeeping genes, and a variable number of alternative σ factors. All alternative σ factors, with the exception of σ^54^, are related to σ^70^.

In *Ec*, transcription at most σ^70^-dependent promoters begins with σ^70^-directed reversible binding of Eσ^70^ to the promoter, which results in the formation of the initial closed promoter complex (RPc). At most promoters, the RPc is unstable and either dissociates or isomerizes via several intermediates to the transcription-initiation-competent open promoter complex (RPo). In the RPo, the promoter DNA strands are locally melted and form a transcription bubble spanning positions −12 to +3 of the promoter and the +1 position of the template strand placed at the RNAp active center (Figure 1A) (Murakami et al., 2002a; Saecker et al., 2011). The double-stranded DNA (dsDNA) immediately downstream of the active center (dwDNA) interacts with a segment of the DBC called the downstream DBC (dwDBC), and this interaction is essential for the formation and stability of the RPo (Murakami et al., 2002a; Saecker et al., 2011). The β′ jaw, β′ insertion 6, and β′ downstream clamp domains contribute to the dwDBC (Murakami et al., 2002a). The dwDNA interaction with the dwDBC can be divided into active center-proximal (+5 to +8) and -distal (+10 to +20) sets of interactions. The active center-distal interactions mainly involve the β′ jaw, β′ insertion 6, and β clamp domains. In the structural model of the RPo, the β′ jaw domain aligns with the path of the dwDNA and is located closest to the dwDNA. The deletion of the β′ jaw domain (amino acid residues 1149–1190) in the *Ec* RNAp dramatically reduces the stability of the RPo (Ederth et al., 2002). Thus, it is very likely, but as yet experimentally unproven, that the β′ jaw domain makes sequence-nonspecific contacts with the dwDNA during RPo formation.

Within the σ^70^ family, only proteins that function as major σ factors contain an extended (∼100 amino-acid-long) amino-terminal domain, called region 1.1 (R1.1). Sequences of R1.1 from major σ factors from various sources are variable but tend to be acidic. At σ^70^-dependent promoters, R1.1 plays an important regulatory role during transcription initiation (see below). Although the solution structure of the isolated domain of R1.1 (amino acid residues 1–100) of *Thermotoga maritima* σ^A^ (a counterpart of *Ec* σ^70^) is available (Schwartz et al., 2008), the R1.1 domain is not resolved in the crystal structures of the σ^A^-containing RNAp from *Thermus aquaticus* and *T. thermophilus* (Murakami et al., 2002a, 2002b; Vassylyev et al., 2002), implying that it is disordered and/or can adopt multiple conformations. Fluorescence resonance energy transfer (FRET) analyses indicate that in the absence of promoter DNA, the position of R1.1 in Eσ^70^ corresponds to that of the dwDNA in the RPo (Mekler et al., 2002). In other words, in Eσ^70^, R1.1 is located deep within the active-center cleft, just above the floor of the dwDBC channel, and is positioned to interact with the floor and walls of the dwDBC (Figure 1A) (Mekler et al., 2002). In the RPo, R1.1 is displaced to the tip of the β-subunit pincer (Mekler et al., 2002). Hence, it has been proposed that in free Eσ^70^, R1.1 acts as a mimic of dwDNA and must be displaced for dwDNA to enter the dwDBC and allow the RNAp active center to access the DNA (Mekler et al., 2002).

Gp2 is a 7 kDa, T7 bacteriophage-encoded, non-DNA-binding transcription factor. The essential biological function of Gp2 is to coordinate transcription of the phage genome by the host and viral RNAps (Savalia et al., 2010). Gp2 binds tightly to the β′ jaw domain of *Ec* RNAp (Cámara et al., 2010; Nechaev and Severinov, 1999) and strongly inhibits transcription from σ^70^-dependent bacterial promoters (Cámara et al., 2010; Nechaev and Severinov, 1999). Gp2 inhibits RPo formation but does not bind to (and therefore has no effect on) preformed RPo (Nechaev and Severinov, 1999). Recently, using an optimized variant of the σ^70^-dependent T5 N25 promoter (hereafter called N25cons), we trapped and characterized a ternary Eσ^70^-Gp2-promoter complex that had normal contacts with promoter DNA upstream of the +1 site and a partially open transcription bubble, but was transcriptionally inactive and lacked the interaction with the dwDNA (Mekler et al., 2011b). Here, we describe the near-atomic resolution solution structure of Gp2 bound to a fragment of the β′ jaw domain (*Ec* β′ subunit residues 1153–1213). We show by nuclear magnetic resonance (NMR) chemical shift perturbation analyses that the β′ jaw domain interacts directly with dsDNA, and that the interaction surface for dsDNA overlaps with the Gp2 binding surface. By using RNAp containing mutant σ^70^ lacking R1.1 (amino acid residues 1–100) and determining the effect of the in *trans* addition of the isolated R1.1 domain on the mutant RNAp activity in the presence of Gp2, we demonstrate that Gp2 inhibits RPo formation by Eσ^70^ not only by sterically preventing dwDNA binding to the dwDBC but also by exerting R1.1-dependent and long-range antagonistic effects on RNAp-promoter DNA interactions near the RNAp active center. Results from biophysical analyses demonstrate that Gp2 changes the microenvironment of R1.1 in Eσ^70^ and restricts the obligatory displacement of R1.1 from the dwDBC during RPo formation. A combination of direct (by competing for dwDNA binding) and indirect (mediated by R1.1) effects makes Gp2 a highly efficient inhibitor of RPo formation by Eσ^70^.

## Results

### Structure of the Complex between Gp2 and the β′ Jaw Domain

A lysine substitution at residue E1158 or E1188 in the β′ jaw domain prevents Gp2 from binding to the *Ec* RNAp (Figure S1A available online) (Cámara et al., 2010; Nechaev and Severinov, 2003). Therefore, the major Gp2 interacting surface on the β′ jaw domain probably includes residues 1158–1188. In agreement with this view, deletion of residues 1149–1190 in the *Ec* RNAp confers resistance to inhibition by Gp2 (Nechaev and Severinov, 1999). We determined the solution structure of the complex between Gp2 and a fragment of the β′ jaw domain (representing *Ec* β′ jaw domain residues 1153–1213; hereafter referred to as the β′ jaw fragment) using multidimensional NMR spectroscopy applied to hybrid-labeled complexes. Backbone C^α^, C^β^, C′, N, and H^N^ assignments for each of the labeled binding partner were obtained from HNCACB/CBCA(CO)NH and HN(CA)CO/HNCO spectra, and side-chain assignments were completed with the use of HCCH total correlation spectroscopy spectra. Broadening of some residues in the binding interface indicates the presence of conformational exchange in the isolated complex, perhaps due to the β′ jaw fragment not fully representing the complete RNAp interaction. Despite this, the interface was initially characterized based on the manual unambiguous assignment of a few intermolecular nuclear Overhauser effect (NOEs) from ^13^C/^15^N-filtered NOE spectroscopy–heteronuclear single quantum coherence (HSQC) spectra. Automated NOE assignment methods using the ARIA program were used to complete the NOE assignment of the complex and calculate a family of 10 structures (Figure S1B and Table 1). The solution structure of the *Ec* β′ jaw fragment consists primarily of a four-stranded antiparallel β sheet (Figures 1B and 1C) and shows high structural similarity to the corresponding region of the β′ subunit in the crystal structures of *T. aquaticus* and *T. thermophilus* RNAp (Figure S1C). In the complex with Gp2, the β sheet is extended to a seven-stranded β sheet in an antiparallel arrangement (Figure 1B). The primary interface region is localized to β3 and β2 of the β′ jaw fragment and β3 and α1 of Gp2. The two invariant arginine residues in Gp2 that are important for binding to the RNAp, R56 and R58 (Cámara et al., 2010), are located in the interface region in close proximity to E1188, providing a significant ionic interaction across the interface (Figure 1B, inset). Additional interfacial residues include L36, L40, T55, and V57 from Gp2, and V1176, Y1186, E1187, E1188, and M1189 from the β′ jaw domain (Figures 1A and 1B, insets). Residues L36, L40, and V57 of Gp2 form the major hydrophobic contacts with V1176 and M1189 of the β′ jaw domain at the binding interface (Figures 1A and 1B, insets). Gp2 contains a contiguous strip of seven negatively charged amino acids (E21, E34, D37, E38, E41, E44, and E53; hereafter referred to as the negatively charged strip [NCS]) on the side of the molecule opposing R56 and R58 (Sheppard et al., 2011). Analyses of the role of the NCS by mutagenesis reveal that the NCS is not important for the binding of Gp2 to RNAp, but the disruption of the NCS significantly attenuates the ability of Gp2 to inhibit RPo formation (Sheppard et al., 2011). An examination of the surface electrostatic properties of the Gp2-β′ jaw fragment complex reveals that the NCS in Gp2 is extended by residues E1158, D1181, D1184, E1187, and E1188 of the β′ jaw domain (Figure S1D).

### Interaction of the β′ Jaw Domain with dsDNA

**Figure 2:**
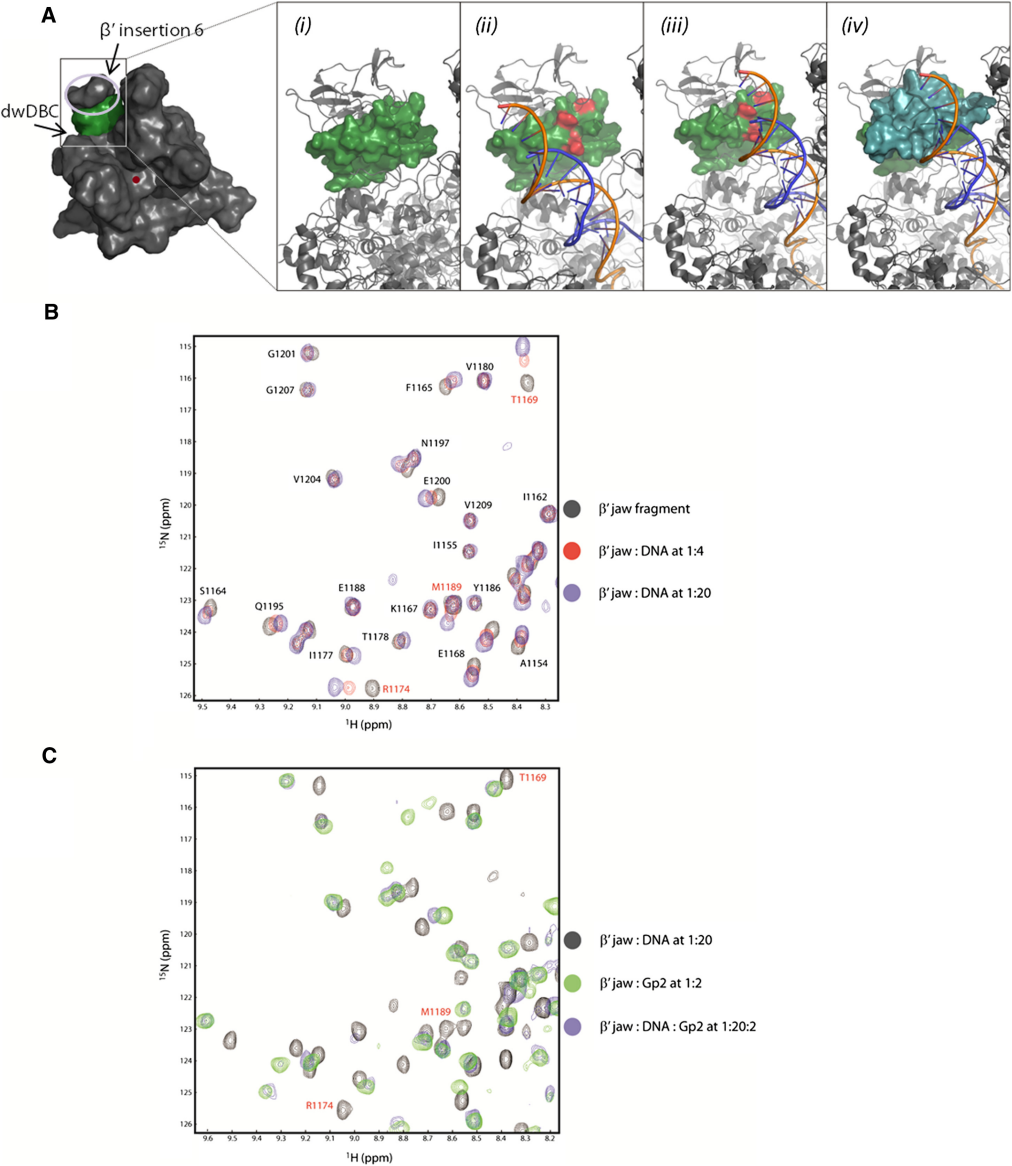
Interaction of the β′ Jaw Domain with dsDNA (A) Surface representation of the *Ec* core RNAp model (Opalka et al., 2010) color-coded as in Figure 1A. The boxed region is enlarged and looks at the DNA-binding surface (shown in ribbon representation in insets i–iv). The β′ jaw domain is shown in green as a surface representation and forms part of the DNA-binding face (i). Inset (ii) is as in (i), but showing the path of the dwDNA from the current model of the RPo (Opalka et al., 2010). Highlighted in red are residues T1169, R1174, and M1189, which undergo significant chemical shift changes in β′ jaw fragment in the presence of dsDNA (B) and Gp2 (C). Inset (iii) is as in (ii), but showing the redefined path of the dwDNA in the dwDBC. Inset (iv) is as in (iii), but with the surface representation of Gp2 shown in cyan. Note the lack of steric clash between Gp2 and the β′ insertion 6 domain, which provides further support for our composite model. (B) Overlay of 2D ^1^H-^15^N HSQC spectra of the β′ jaw fragment with and without dsDNA recorded at pH 6.5, 303 K (see key for details). Peaks with significant chemical shift differences are indicated in red with their residue numbers (T1169, R1174, and M1189). (C) As in B, but showing the 2D ^1^H-^15^N HSQC spectra of the β′ jaw fragment with dsDNA (i.e., the β′ jaw fragment is ^15^N labeled) with or without unlabeled Gp2 (see key for details).

To better understand how Gp2 inhibits RPo formation by Eσ^70^, we derived a composite structural model of the Gp2-RNAp complex using our solution structure of the Gp2-β′ jaw fragment complex together with structural models of the *Ec* core RNAp (Opalka et al., 2010) and RPo based on the structure of the *T. aquaticus* RNAp (Murakami et al., 2002a). In the composite structural model, the Gp2-binding surface of the β′ jaw domain is facing the dwDBC and toward where the dwDNA would likely lie in the RPo (Figure 2A, insets *i* and *ii*). Because Gp2 antagonizes the interaction between the dwDNA and dwDBC during RPo formation (Mekler et al., 2011a, 2011b), we hypothesized that this region of the β′ jaw domain would make sequence-nonspecific direct contacts with dsDNA, and the binding of Gp2 could block or modulate its interaction with dsDNA. To test whether an interaction exists between the β′ jaw domain and dsDNA during RPo formation, we conducted an NMR titration experiment with a randomly generated 14 bp dsDNA fragment and ^15^N-labeled β′ jaw fragment. We recorded 2D ^1^H-^15^N HSQC spectra to monitor the backbone amide chemical shift changes in the β′ jaw fragment in the presence of DNA. The NMR spectrum exhibited several specific chemical shift changes, which were in fast exchange on the NMR timescale indicative of a binding constant in the micromolar to millimolar range (Figure 2B). The major perturbed residues (T1169, R1174, and M1189) map to the exposed surface of the β3 sheet of the β′ jaw fragment (Figure 2A, inset *ii*, and Figure S1A), suggesting that these residues are involved in interaction with dsDNA. Consistent with this view, results from formaldehyde crosslinking experiments showed that whereas the wild-type β′ jaw fragment could be crosslinked to the ^32^P-labeled 14 bp dsDNA, a mutant β′ jaw fragment containing an alanine substitution at R1174 could not be detectably crosslinked to the dsDNA (Figure S1E, compare lanes 2 and 4). Further, the R1174A mutation in the context of Eσ^70^ formed a significantly reduced number of RPo compared with the wild-type Eσ^70^ (Figure S1F). However, once the RPo were formed, the stabilities of the mutant and wild-type RPo upon challenge with heparin were indistinguishable (Figure S1F), which is not surprising considering that the interface between Eσ^70^ and DNA is extensive in the RPo, and the effect of a single point mutation in the β′ jaw domain on overall DNA binding by Eσ^70^ would be difficult to detect. Thus, in the composite model, we redefined the potential path of the dsDNA in the RPo with respect to the β′ jaw domain (Figure 2A, inset *iii*). A comparison of the DNA-interacting surface in the β′ jaw fragment with the Gp2-binding interface from our solution structure of the Gp2-β′-fragment complex reveals significant overlap and suggests that Gp2 and dsDNA may compete for overlapping interaction surfaces on the β′ jaw domain in the RNAp (Figure 2A, inset iii and iv). To test this hypothesis, we performed a competition NMR experiment by titrating Gp2 into a saturated complex of the β′ jaw-fragment-dsDNA complex and recorded the changes in the NMR spectrum. As shown in Figure 2C, the characteristic NMR spectrum of the Gp2-β′ jaw-fragment complex was regained after the addition of Gp2, thus confirming that in the context of the isolated β′ jaw fragment, Gp2 is able to displace dsDNA efficiently. Consistent with this view, results from formaldehyde crosslinking experiments showed that in the presence of Gp2, the β′ jaw fragment could not be efficiently crosslinked to the ^32^P-labeled 14 bp dsDNA (Figure S1E, compare lanes 2 and 3). Overall, the results strongly suggest that Gp2 and DNA compete for overlapping binding sites on the β′ jaw domain in the RNAp, and provide a structural basis for and further insights (see below) into the mechanism by which Gp2 inhibits RPo formation by Eσ^70^.

### Gp2 Requires R1.1 to Efficiently Inhibit RPo Formation by Eσ^70^

**Figure 3:**
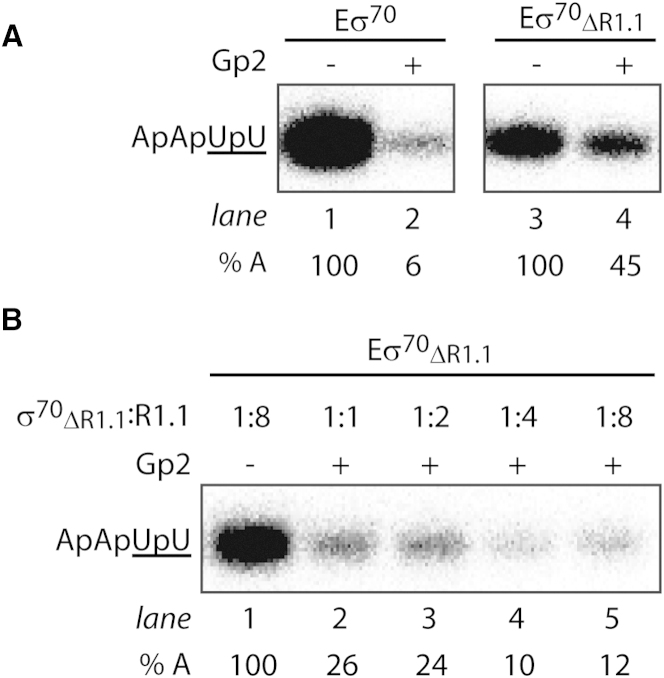
Gp2 Requires R1.1 to Efficiently Inhibit RPo Formation by Eσ^70^ (A) Autoradiograph of 20% (v/v) denaturing urea gels showing the synthesis of the ApApUpU transcript (underlined nucleotides are α^32^P labeled) from the *lac*UV5 promoter by Eσ^70^ (lanes 1 and 2) and Eσ^70^_ΔR1.1_ (lanes 3 and 4) in the absence and presence of Gp2. The percentage of ApApUpU transcript synthesized (% A) in the reactions with Gp2 with respect to reactions with no Gp2 is given at the bottom of the gel for each reaction. (B) As above, but showing the synthesis of the ApApUpU transcript in the absence (lane 1) and presence (lanes 2–5) of Gp2 under conditions in which Eσ^70^_ΔR1.1_ was preincubated with increasing amounts of isolated R1.1 domain added in *trans* to the reaction (shown as the ratio of σ^70^_ΔR1.1_ to R1.1). For (A) and (B), all data obtained in at least three independent experiments fell within 5% of the % A value shown. See also Figure S2.

The location of Gp2 in our composite structural model of the Gp2-RNAp complex places Gp2 proximal to the location of R1.1 of σ^70^ in Eσ^70^ inferred from biophysical studies (Mekler et al., 2002). Thus, it is possible that Gp2 could affect R1.1 function during RPo formation, and therefore Gp2 could inhibit RPo formation by a mechanism involving R1.1 of σ^70^. To test this hypothesis, we explored the role of R1.1 in the mechanism by which Gp2 inhibits RPo formation by Eσ^70^. Initially, we determined the ability of Gp2 to inhibit Eσ^70^ reconstituted with either wild-type σ^70^ or σ^70^_ΔR1.1_ using an in vitro transcription assay. Incubation of an ∼2-fold molar excess of Gp2 with Eσ^70^ before the addition of a DNA fragment containing the *lac*UV5 promoter abolished the synthesis of *lac*UV5-specific ApApUpU transcript (Figure 3A, lanes 1 and 2) (Cámara et al., 2010). In contrast, under identical conditions, Eσ^70^_ΔR1.1_ was inhibited far less efficiently (∼55% inhibition; Figure 3A, lanes 3 and 4) even though the affinity of Gp2 for Eσ^70^_ΔR1.1_ and Eσ^70^ did not differ detectably among the conditions under which the in vitro transcription assays were performed (Figures S2A and S2B). Therefore, it seems that full inhibition of Eσ^70^ RPo formation by Gp2 requires R1.1 of σ^70^. Consistent with this view, the in *trans* addition of the isolated domain of σ^70^ R1.1 (encompassing σ^70^ amino acids 1–100) to Eσ^70^_ΔR1.1_ increased the efficiency of Eσ^70^_ΔR1.1_ transcription inhibition by Gp2, bringing it to the same level as in the case of Eσ^70^ (Figure 3B, lanes 2–5). Thus, the presence of the isolated R1.1 domain in *trans* in Eσ^70^_ΔR1.1_ allows Gp2 to efficiently inhibit RNAp. Full inhibition of Eσ^70^_ΔR1.1_ by Gp2 occurred when the isolated R1.1 domain was added in *trans* to the Eσ^70^_ΔR1.1_ either before or after Gp2 binding (Figure S2C). Control reactions established that the in *trans* presence of the isolated R1.1 domain in Eσ^70^_ΔR1.1_ (in the absence of Gp2) did not antagonize the ability of Eσ^70^_ΔR1.1_ to synthesize the ApApApU transcript (Figure S2D).

An alternative *Ec* σ factor, σ^38^, does not contain R1.1 but is able to recognize some σ^70^-dependent promoters (Gruber and Gross, 2003). We compared the ability of Gp2 to inhibit RPo formation by Eσ^38^ and Eσ^70^ on one such promoter, the *Ec osmE* promoter (Bordes et al., 2000). Although the affinity of Gp2 for Eσ^38^ and Eσ^70^ did not differ detectably among the conditions under which the in vitro transcription assays were performed (Figures S2A and S2B), Gp2 inhibited Eσ^38^-dependent synthesis of the ApApCpA *osmE* transcript by only ∼80% even under conditions in which the amount of Gp2 exceeded that of Eσ^38^ by ∼4-fold (Figure S2E). In contrast, transcription initiation by Eσ^70^ from this promoter was barely detectable under the same conditions (Figure S2E). As expected, full inhibition of RPo formation by Eσ^70^ on the *osmE* promoter was R1.1 dependent (Figure S2F, lanes 3–6). Moreover, in the presence of the R1.1 domain of σ^70^ added in *trans*, Gp2 fully inhibited RPo formation by Eσ^38^ on the *osmE* promoter (Figure S2F, lanes 7–10). In summary, even though no detectable differences in the affinity of Gp2 for Eσ^70^, Eσ^70^_ΔR1.1_, and Eσ^38^ were observed in two independent experiments (Figures S2A and S2B), we cannot exclude the possibility that the absence of R1.1 (as in the case of Eσ^70^_ΔR1.1_ and Eσ^38^) can affect the affinity of Gp2 for RNAp. However, the results strongly suggest that R1.1 of σ^70^ is part of the mechanism by which Gp2 inhibits RPo formation by Eσ^70^: Gp2 alone antagonizes dwDNA binding to the dwDBC, leading to partial inhibition of RPo formation, and full inhibition requires R1.1 of σ^70^.

### Gp2 Requires R1.1 of σ^70^, but Not the Consensus Promoter DNA Sequences, to Fully Inhibit RPo Formation by Eσ^70^

**Figure 4:**
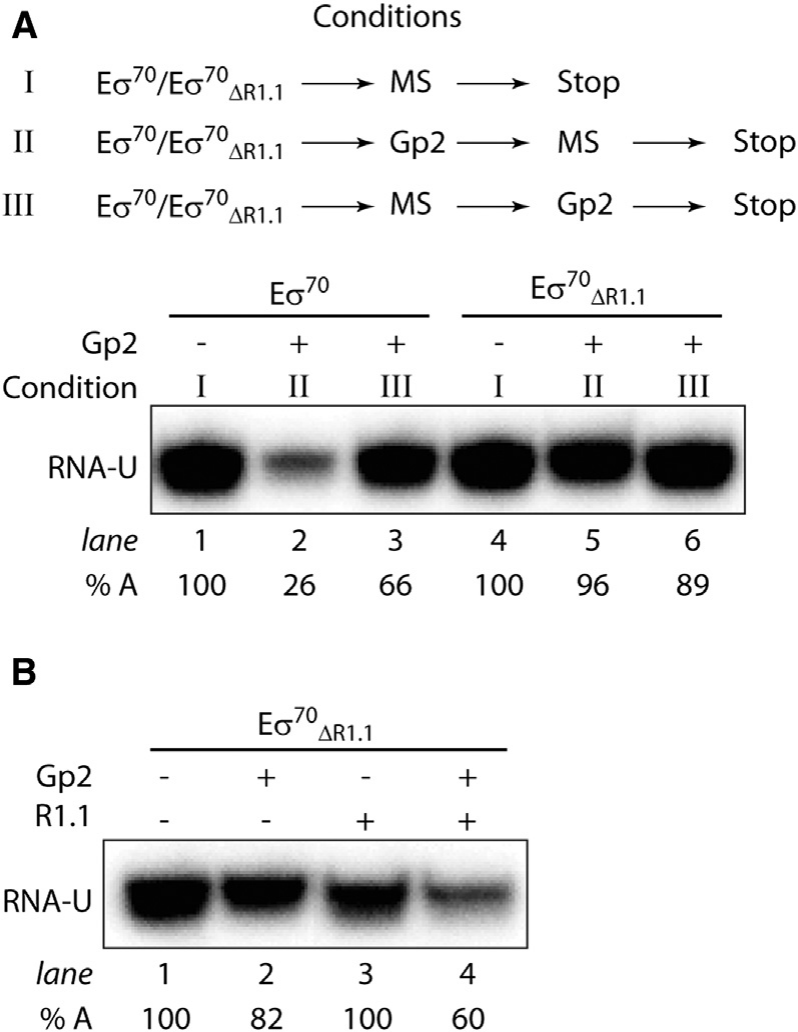
Gp2 Requires R1.1 of σ^70^, but Not the Consensus Promoter DNA Sequences, to Fully Inhibit RPo Formation by Eσ^70^ (A) Autoradiograph of a 20% (v/v) denaturing urea gel showing the synthesis of RNA-U from the MS probe by Eσ^70^ and Eσ^70^_ΔR1.1_ in the absence (condition I) and presence (conditions II and III) of Gp2. The percentage of RNA-U synthesized (% A) in the reactions with Gp2 with respect to reactions with no Gp2 is given at the bottom of the gel for each reaction. (B) As in (A), except that the reaction was conducted with Eσ^70^_ΔR1.1_ in the absence and presence (at ∼8-fold molar excess over σ^70^_ΔR1.1_) of isolated R1.1 domain added in *trans*. For (A) and (B), all data obtained in at least three independent experiments fell within 5% of the % A value shown. See also Figure S3.

The σ^70^ factor makes extensive contacts with the consensus promoter DNA sequences (i.e., the −35 and −10 promoter elements) in the RPc and during RPo formation. Because full inhibition of Eσ^70^ RPo formation by Gp2 depends on R1.1 of σ^70^, we considered whether interactions between Eσ^70^ and the consensus promoter DNA sequences play any role in the mechanism by which inhibition of RPo formation by Gp2 at σ^70^-dependent promoters occurs. To address this issue experimentally, we determined whether Gp2 could inhibit the catalytic activity of Eσ^70^ on a promoterless minimal nucleic acid scaffold template (hereafter called the minimal scaffold [MS] probe). The MS probe consists of an 18-nucleotide-long DNA duplex and an 8-nucleotide-long RNA-DNA heteroduplex separated by two unpaired DNA bases (Kulbachinskiy et al., 2004) (Figure S3A). Thus, the MS probe lacks the consensus promoter DNA sequences recognized by σ^70^. The addition of α^32^P-UTP to the Eσ^70^-MS probe complex results in the synthesis of a nine-nucleotide-long α^32^P-UTP-labeled RNA product, hereafter called RNA-U (Kulbachinskiy et al., 2004). As shown in Figure 4A, lanes 1 and 2, Gp2 inhibits the synthesis of RNA-U from the Eσ^70^-MS probe complex by preventing Eσ^70^ from binding to the MS probe (see also Figure S3B, lanes 1–4). In contrast, and as expected, the addition of Gp2 to the preformed Eσ^70^-MS probe complex had relatively little effect on the amount of RNA-U synthesized (Figure 4A, lane 3). Under identical conditions, when Eσ^70^_ΔR1.1_ was used, no inhibition of RNA-U synthesis by Gp2 was observed (Figure 4A, lanes 4–6). The in *trans* addition of the isolated R1.1 domain to Eσ^70^_ΔR1.1_ conferred a significant degree of Gp2 sensitivity upon the Eσ^70^_ΔR1.1_-dependent transcription from the MS probe (Figure 4B, lane 4). Further, in the absence of σ^70^, the catalytic activity of core RNAp on the MS probe was unaffected by Gp2 (Figure S3C). In summary, the results obtained with the MS probe corroborate the view that full inhibition of Eσ^70^ RPo formation by Gp2 depends on σ^70^ (specifically the R1.1 domain) but occurs independently of the consensus promoter DNA elements.

### Inhibition of RPo Formation by Gp2 Involves a Long-Range, R1.1-Dependent, Antagonistic Effect on Eσ^70^-Promoter Interactions

**Figure 5:**
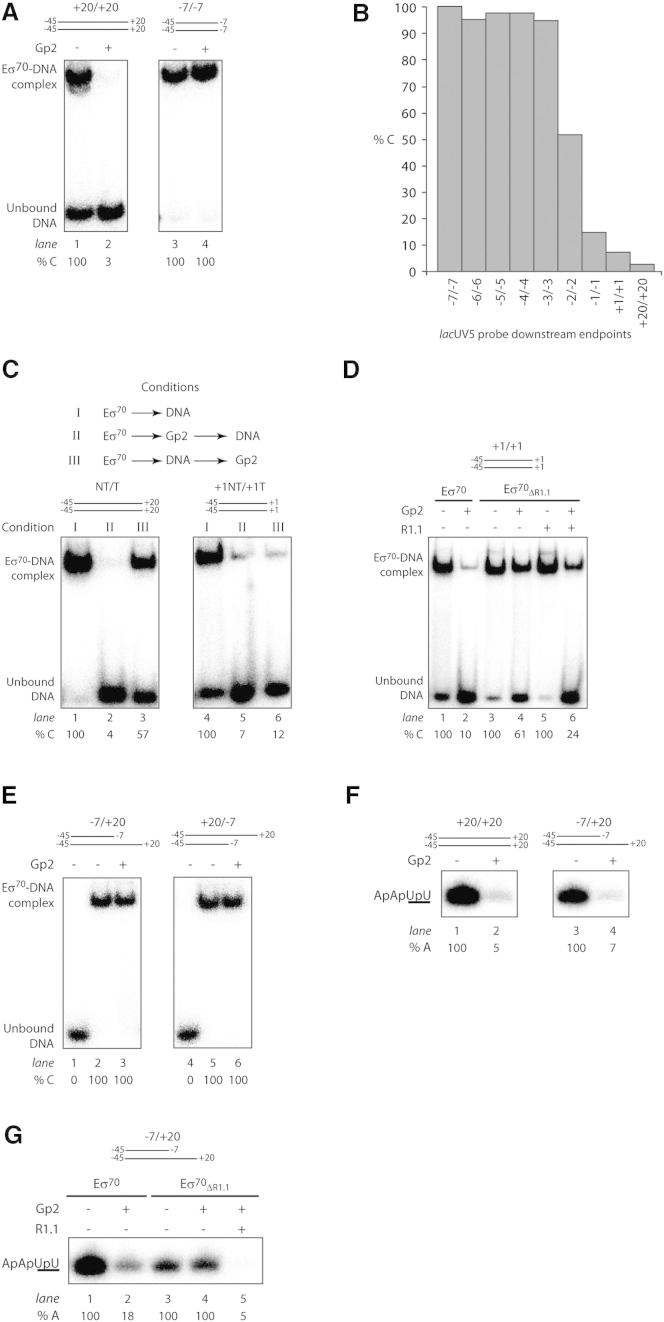
Inhibition of RPo Formation by Gp2 Involves Long-Range Antagonistic Effects on Eσ^70^-Promoter Interactions (A) Autoradiograph of a 4% (v/v) native polyacrylamide gel comparing binding of Eσ^70^ to the +20/+20 (lanes 1 and 2) and −7/−7 (lanes 3 and 4) probes in the presence (lanes 2 and 4) and absence (lanes 1 and 3) of Gp2. (B) Graph showing the percentage of DNA bound by Eσ^70^ in the presence of Gp2 compared with reactions with no Gp2. The dsDNA probes with different downstream end points are indicated in the *x* axis of the graph. (C) Autoradiograph of 4% (v/v) native polyacrylamide gels comparing binding of Eσ^70^ with the +20/+20 (lanes 1–3) and +1/+1 (lanes 4–6) probes in the absence (condition I) and presence (conditions II and III) of Gp2. (D) Autoradiograph of a 4% (v/v) native polyacrylamide gel comparing binding of Eσ^70^ (lanes 1 and 2) and Eσ^70^_ΔR1.1_ (lanes 3–6) in the presence (lanes 2, 4, and 6) and absence (lanes 1, 3, and 5) of Gp2 to the +1/+1 probe. In lanes 5 and 6, the isolated R1.1 domain is present in *trans*. (E) Autoradiograph of 4% (v/v) native polyacrylamide gels comparing binding of Eσ^70^ to promoter probes with either the nontemplate or template strand ending at the −7 position (−7/+20 and +20/−7) in the absence (lane 2 and 5) and presence (lanes 3 and 6) of Gp2. In (A–E), the percentage of DNA bound by Eσ^70^ or Eσ^70^_ΔR1.1_ (% C) in the reactions with Gp2 with respect to reactions with no Gp2 is given at the bottom of the gels. The data obtained in at least two independent experiments fell within 3% of the % C value shown. (F) Autoradiographs of 20% (v/v) denaturing urea gels showing the synthesis of the ApApUpU transcript (underlined nucleotides are α^32^P labeled) from the *lac*UV5 promoter +20/+20 and −7/+20 probes by Eσ^70^ in the absence (lanes 1 and 3) and presence (lanes 2 and 4) of Gp2. (G) As in F, but using the −7/+20 probe comparing the activity of Eσ^70^ and Eσ^70^_ΔR1.1_ in the absence (lanes 1 and 3) and presence (lanes 2, 4, and 5) of Gp2. In lane 5, the isolated R1.1 domain is present in *trans* (at ∼8-fold molar excess over σ^70^_ΔR1.1_). In (F) and (G), the percentage of ApApUpU transcript synthesized (% A) in the reactions with Gp2 with respect to reactions with no Gp2 is given at the bottom of the gel for each reaction. The data obtained in at least two independent experiments fell within 5% of the % A value shown. See also Figure S4.

In the RPc, the promoter DNA does not interact with the dwDBC, and consistent with previous results, RPc formation is not inhibited by Gp2 (Cámara et al., 2010; Mekler et al., 2011b). We next conducted experiments to investigate whether the binding of Gp2 to the β′ jaw domain influences RNAp-promoter interaction outside of the dwDBC during RPo formation, and determine what role (if any) R1.1 plays in this process. We conducted electrophoretic gel mobility shift assays (EMSAs) to determine whether Eσ^70^ binding to shortened versions of the σ^70^-dependent *lac*UV5, λP_R3_, and T7A1 promoter probes truncated at position −7 of both strands (the −7/−7 probes) is inhibited by Gp2. Note that the −7/−7 probes contain the −35 and −10 consensus promoter DNA elements recognized by σ^70^ regions 4.2 and 2.4, respectively, and lack the dwDNA segment. The results reveal that the binding of Eσ^70^ to the −7/−7 probes is not inhibited by Gp2 (Figure 5A, lanes 3 and 4, and Figure S4A), and are thus consistent with the view that interactions between the RNAp and the promoter in the RPc are not affected by Gp2. In contrast, under identical conditions, the binding of Eσ^70^ to the corresponding +20/+20 probes is abolished by Gp2, as expected (Figure 5A, lanes 1 and 2, and Figure S4A).

We next determined the minimum length of the promoter template at which inhibition by Gp2 starts to occur. To that end, we extended the *lac*UV5 −7/−7 probe in one-basepair increments and monitored the Gp2 sensitivity of complex formation with these probes by EMSA. The results show that binding of Eσ^70^ to the −2/−2 probe is reduced by 50% in the presence of Gp2, and binding of Eσ^70^ to the −1/−1 and +1/+1 probes is inhibited by 85% and 100%, respectively (Figure 5B and Figure S4B). Because the site of Gp2 binding, the β′ jaw, is located downstream of the +1 position, the inhibitory effect of Gp2 evidently extends beyond the inhibition of dwDNA interactions with the β′ jaw during RPo formation (see above). In support of the above view, whereas inhibition of Eσ^70^ binding to the +20/+20 DNA depends on the order of addition, Gp2 inhibited the binding of Eσ^70^ to the +1/+1 probe independently of the order of addition (Figure 5C; compare lanes 1–3 and 4–6). The order of addition-independent inhibitory effect of Gp2 on the binding of Eσ^70^ to the +1/+1 probe is specific, because the binding of Eσ^70^ to the +1/+1 probe is not inhibited by an RNAp-binding mutant of Gp2 (R56E) (Cámara et al., 2010) (Figure S4C). The binding of Gp2 to the β′ jaw per se was not the cause of inhibition of Eσ^70^ binding to the +1/+1 probe, because Eσ^70^ bound the +1/+1 probe in the presence of a Gp2 mutant (Mut7) that binds RNAp normally but is functionally attenuated (Sheppard et al., 2011) (Figure S4C). Furthermore, the inhibitory effect of Gp2 was markedly reduced when RNAp containing σ^70^ lacking R1.1 was used to bind the +1/+1 probe (Figure 5D, lanes 3 and 4) and, as expected, was partially restored when the isolated R1.1 domain was added in *trans* to this reaction (Figure 5D, lanes 5 and 6). Overall, we conclude that inhibition of the RPo formation by Gp2 also involves a long-range, R1.1-dependent antagonistic effect on Eσ^70^ interactions with DNA around the RNAp active center.

### Gp2 Interferes with the Promoter DNA Template Strand Accessing the RNAp Active Center

We next wanted to determine the effect of Gp2 on Eσ^70^ binding to variants of the −7/−7 *lac*UV5 probe with either template or nontemplate single-strand downstream extensions to position +20 (−7/+20 or +20/−7 promoter probes, respectively; recall that RPo formation on the +20/+20 probe is efficiently inhibited by Gp2). The EMSA results reveal that Gp2 had no detectable effect on the binding of Eσ^70^ to either of these probes (Figure 5E). To determine whether the RNAp active site can access the +1 position on the template strand of the −7/+20 probe in the presence of Gp2, we performed transcription-initiation assays. The results, shown in Figure 5F, indicate that even though Gp2 does not inhibit the binding of Eσ^70^ to this probe (Figure 5E, lanes 1–3), the synthesis of the ApApUpU transcript is effectively abolished in the presence of Gp2. The inhibitory effect of Gp2 on transcription from the −7/+20 probe is R1.1 dependent: ApApUpU transcript synthesis by Eσ^70^_ΔR1.1_ is not inhibited by Gp2 (Figure 5G, lanes 3 and 4), whereas the in *trans* addition of the isolated R1.1 domain to Eσ^70^_ΔR1.1_-containing reactions abolishes ApApUpU synthesis (Figure 5G, lane 5). Therefore, we conclude that the RNAp active center cannot productively access the template strand of the −7/+20 promoter probe when Gp2 is bound to the β′ jaw domain. This conclusion is consistent with the view that the binding of Gp2 to the β′ jaw has a long-range, R1.1.-mediated antagonistic effect on Eσ^70^-promoter interactions near the RNAp active center. In other words, it seems that the binding of Gp2 to the β′ jaw in Eσ^70^ restricts single-stranded DNA from accessing the RNAp active center in an R1.1-dependent manner.

### Gp2 Appropriates R1.1 to Efficiently Inhibit RPo Formation by Eσ^70^

**Figure 6:**
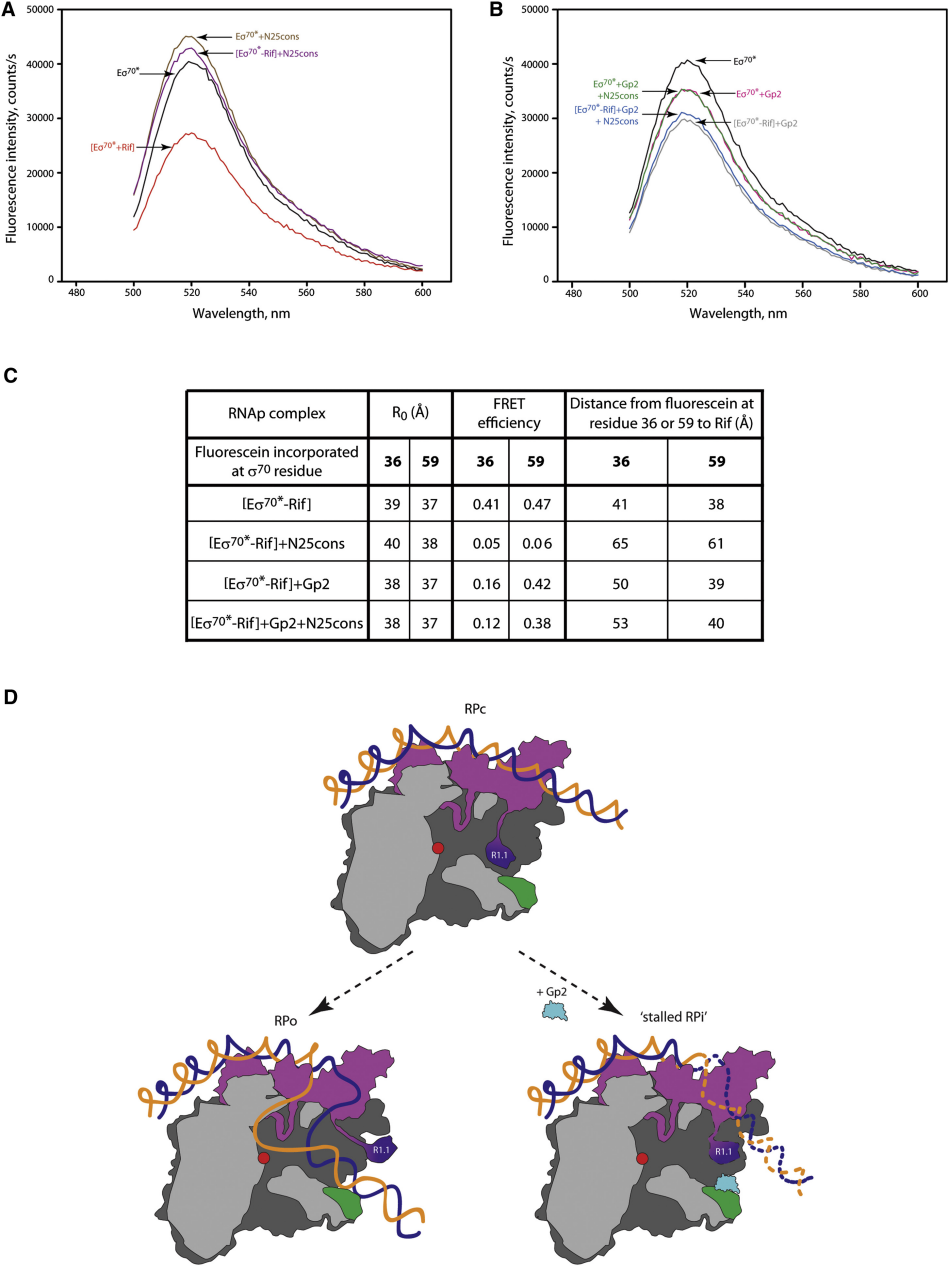
Gp2 Appropriates R1.1 to Efficiently Inhibit RPo Formation by Eσ^70^ (A and B) Measurement of FRET between fluorescein incorporated into σ^70^ at position 36 (σ^70∗^) and Rif during RPo formation in the absence (A) and presence (B) of Gp2. The fluorescence emission spectra are recorded with 482 nm excitation. (C) The FRET efficiency values and distance calculations are tabulated (see also Figure S5) and the values presented are averages obtained from two to three individual experiments; the estimated error in R_0_ is ∼10%. (D) Cartoon (as in Figure 1A) depicting the mechanism by which RPo formation is inhibited by Gp2 at σ^70^-dependent promoters.

Previously, Mekler et al. (2002) showed that FRET can be used to monitor the displacement of R1.1 from near the dwDBC in free Eσ^70^ to the tip of the β pincer in the RPo (see Introduction). Because Gp2 efficiently inhibits transcription initiation in an R1.1-dependent manner, we wanted to determine whether Gp2 antagonizes the obligatory displacement of R1.1 during RPo formation. Initially, we calculated the distance between R1.1 and the RNAp active center by measuring FRET between a fluorescein probe incorporated at amino acid position 36 in σ^70^ R1.1 (hereafter called σ^70∗^) and rifampicin (Rif), an antibiotic that binds RNAp between the upstream and downstream lobes of the β subunit and effectively quenches the fluorescence in Eσ^70∗^ (Figure S5A and Figure 6A; compare lines labeled Eσ^70∗^ and [Eσ^70∗^-Rif]). As expected, the quenching efficiency is much lower in the RPo formed on the N25cons promoter than with Eσ^70∗^ (Figure 6A; compare lines labeled Eσ^70∗^ and [Eσ^70∗^+Rif] with lines labeled Eσ^70∗^+N25cons and [Eσ^70∗^-Rif]+N25cons, respectively), thus indicating displacement of R1.1 from near the dwDBC upon RPo formation. In control experiments in which Rif was replaced with the colorless RNAp inhibitor sorangicin-A (Sor), which binds RNAp in the Rif-binding site (Campbell et al., 2005), no influence on the fluorescence of the fluorescein probe attached to position 36 in R1.1 was detected (Figure S5B; compare lines Eσ^70∗^ and [Eσ^70∗^-Sor]). Further, the addition of Rif to the preformed Eσ^70∗^+Sor complex also resulted in a negligible decrease in fluorescence intensity (Figure S5B; compare lines [Eσ^70∗^-Sor] and [Eσ^70∗^-Sor]+Rif). Thus, the Rif-mediated decrease in fluorescence intensity of the fluorescein probe attached to amino acid position 36 in R1.1 is specific to the binding of Rif and is a consequence of quenching via the FRET mechanism. The addition of Gp2 caused an ∼13% decrease in the fluorescence intensity of Eσ^70∗^ (Figure 6B; compare lines labeled Eσ^70∗^ and Eσ^70∗^+Gp2), suggesting that the binding of Gp2 to the β′ jaw domain changes the microenvironment of R1.1 and/or its positions with respect to the RNAp active center. These effects result from specific Gp2 binding to the β′ jaw domain, because no change in fluorescence spectra was observed in the presence of the R56E Gp2 mutant (Sheppard et al., 2011), which does not bind to the RNAp (compare Figure S5C and Figure 6B). The calculated distances between the fluorescein probe attached to amino acid position 36 in R1.1 and Rif are 41 Å and 65 Å in Eσ^70∗^ and RPo, respectively (Figure 6C). However, in the [Eσ^70∗^-Rif]+Gp2 complex, this distance is longer than in the [Eσ^70∗^-Rif] complex by 9 Å (Figure 6C). When the N25cons probe is added to the [Eσ^70∗^-Rif]+Gp2 complex, the calculated distance between fluorescein at amino acid position 36 in R1.1 and Rif is 53 Å (Figure 6B, compare lines labeled [Eσ^70∗^-Rif]+Gp2 and [Eσ^70∗^-Rif]+Gp2+N25cons, and Figure 6C). In contrast, as mentioned above, when the N25cons probe is added to the [Eσ^70∗^-Rif] complex (i.e., in the absence of Gp2), the corresponding calculated distance is 65 Å (Figure 6A, compare lines labeled [Eσ^70∗^-Rif] and [Eσ^70∗^-Rif]+N25cons, and Figure 6C). Thus, Gp2 reduces the distance of R1.1 displacement during RPo formation by 12 Å. This effect is specific, because no change in fluorescence spectra was observed in control experiments with R56E Gp2 mutant (compare Figure S5C and Figure 6B). Because Eσ^70^ forms a complex with the N25cons promoter, which contains Gp2 and bears some hallmarks of the RPo formed in the absence of Gp2 (see Introduction), our results indicate that ternary (Eσ^70^-Gp2-N25cons promoter) complex formation is not accompanied by the characteristic long-distance displacement of R1.1 that normally occurs during RPo formation (Figures 6A and 6C). To avoid a possible error related to uncertainty of the Rif-fluorescein distance determination (Knight et al., 2005), we performed control experiments conducted with fluorescein probe attached to a different position in R1.1 (amino acid 59), which further corroborated our conclusion (Figure 6C and Figure S5E). When the N25cons probe is added to the [Eσ^70^59^∗^-Rif]+Gp2 complex, the calculated distance between the fluorescein at amino acid position 59 in R1.1 and Rif is 40 Å (Figure S5F, compare lines labeled [Eσ^70^59^∗^-Rif]+Gp2 and [Eσ^70^59^∗^-Rif]+Gp2+N25cons, and Figure 6C). In contrast, when the N25cons probe is added to the [Eσ^70^59^∗^-Rif] complex (i.e., in the absence of Gp2), the corresponding calculated distance is 61 Å (Figure S5E, compare lines labeled [Eσ^70^59^∗^-Rif] and [Eσ^70^59^∗^-Rif]+N25cons, and Figure 6C). Thus, Gp2 appropriates R1.1 to efficiently inhibit RPo formation by Eσ^70^.

## Discussion

The interaction between dwDNA and dwDBC in the RNAp is important for establishing the RPo for transcription initiation at bacterial promoters. The *Ec* RNAp β′ jaw domain, a pivotal feature of the dwDBC, is a multifunctional domain whose role in transcription extends beyond RPo formation (Ederth et al., 2006). In previous studies, Ederth et al. (2006) and our group (Wigneshweraraj et al., 2006) showed that the β′ jaw domain is involved in regulatory interplay with other parts of the RNAp that extend to the distally located nascent RNA-binding site. In vitro, deletion of the β′ jaw domain in the *Ec* RNAp destabilizes RPo, suppresses transcriptional pausing, increases the overall elongation rate, and decreases intrinsic termination (Ederth et al., 2002, 2006). Here, we have demonstrated that the β′ jaw domain makes sequence-nonspecific contacts with dsDNA. *Ec* RNAp mutants with the G1161R mutation or the deletion of residues 1149–1190 in the β′ jaw domain form RPo with significantly reduced half-lives compared with RPo formed by the wild-type RNAp. Our data provide an explanation for the observed phenotypes of the mutant RNAp in a structural context: (1) the β′ jaw domain residues that undergo significant chemical shift changes upon interaction with dsDNA (T1169, R1174, and M1180) are all located within the deleted region (i.e., 1149–1190), and (2) the invariant G1161 residue (Figure S1A) is located in the β1 sheet of the β′ jaw domain structure and is facing away from the DNA-binding surface toward the β′ insertion 6 domain; thus, an arginine side chain at this position could compromise the overall structural integrity of the dwDBC. During T7 phage infection of *Ec*, the sequence-nonspecific interaction between the β′ jaw domain and dsDNA is subject to regulation by the small, non-DNA-binding T7 transcription factor Gp2, which inhibits RPo formation by the host RNAp. One strategy used by Gp2 to inhibit RPo formation by the host RNAp is to sterically occlude dsDNA from binding to the β′ jaw domain by competing with dsDNA for overlapping interaction surfaces on the β′ jaw domain.

The narrow width of the dwDBC observed in structures of the bacterial RNAp is thought to constrain the entry of DNA into the catalytic cleft of RNAp for RPo formation. An obligatory step during RPo formation at σ^70^-dependent promoters is the displacement of the R1.1 domain of σ^70^ from near the dwDBC to the tip of the β pincer, which occurs concomitantly with the loading of DNA in the DBC/dwDBC. The negatively charged R1.1 domain is believed to act as a molecular placeholder for dsDNA in Eσ^70^; therefore, the displacement of R1.1 would facilitate the loading of dwDNA into the dwDBC and subsequently stabilize the formation of RPo (Mekler et al., 2002). Thus, it was proposed that R1.1 can facilitate DNA entry into the dwDBC by holding the β and β′ pincers open so that the promoter DNA can enter the dwDBC and the template promoter strand can access the RNAp active center (Saecker et al., 2011). Our results demonstrate that R1.1 of σ^70^ has an important functional role in the mechanism by which Gp2 inhibits RPo formation at σ^70^-dependent promoters. Consistent with this view, Gp2 inhibits RPo formation by RNAp containing alternative σ factors such as σ^38^ or σ^54^ less efficiently or not detectably, respectively (see above) (Wigneshweraraj et al., 2004). We envisage a model in which Gp2 repositions R1.1 (see below) and/or stabilizes R1.1 so that it can no longer be displaced to the tip of the β pincer. This would allow RPo to form and thus stall the Eσ^70^-promoter complex in an intermediate state (intermediate promoter complex [RPi] in Figure 6D) at σ^70^-dependent promoters. Furthermore, the extended negatively charged patch in the dwDBC that results upon binding of Gp2 to the β′ jaw domain could thus help electrostatically reposition R1.1 and/or mimic the presence of R1.1 in the dwDBC. In support of the former view, the removal of the negatively charged side chains of the amino acids in Gp2 that contribute to the NCS does not affect the affinity of Gp2 to the RNAp or its overall structural integrity, but it does severely decrease its ability to inhibit RPo formation (Sheppard et al., 2011). Intriguingly, the R1.1-dependent inhibitory effect of Gp2 on the binding of dwDNA to the β′ jaw domain extends beyond the dwDBC and has long-range antagonistic effects on Eσ^70^-promoter interactions that extend up to the RNAp active center. As a consequence, Eσ^70^-promoter DNA interactions become stalled en route to the RPo (Figure 6D). Because not even single-stranded DNA can access the active center of the RNAp in the presence of Gp2, it seems that Gp2 when bound to the β′ jaw domain restricts the conformational flexibility and changes in the RNAp that normally accompany RPo formation. Because the interaction between Gp2 and the isolated β′ jaw domain fragment is several orders of magnitude weaker than that between Gp2 and the whole enzyme, we cannot exclude the possibility that Gp2 undergoes positional rearrangements and makes additional contacts with the other domains of the RNAp that surround the dwDBC, notably the β′ insertion 6 and/or the β downstream lobe domains. It is therefore likely that the solution structure of the Gp2-β′ jaw fragment likely reports on an early encounter complex between Gp2 and RNAp.

The biological role of Gp2 is to inhibit the transcription of early T7 genes from strong σ^70^-dependent promoters present on the T7 genome by the host RNAp (Savalia et al., 2010). The absence of Gp2 results in unsuccessful infection because the antiterminated host RNAp moves into regions of the T7 genome that are normally transcribed by the T7 RNAp. The interference of the fast-moving T7 RNAp, which is responsible for the transcription of middle and late T7 genes, by the slow-moving host RNAp results in aberrant packaging of concatemeric viral DNA into virion heads, and thus unsuccessful infection. The two-pronged strategy used by Gp2 to inhibit the host RNAp, through occlusion of dwDNA from binding to the dwDBC and appropriation of a σ^70^-specific domain, leads to highly efficient inhibition of Eσ^70^-dependent transcription from very strong early T7 promoters to ensure successful infection. The inhibition of host RNAp by Gp2 thus defines an as yet uncharacterized mechanism by which bacterial transcription is regulated by a nonbacterial factor.

## Experimental Procedures

### Proteins and Promoter Templates

Details of the proteins and promoter templates used in this study are provided in the Supplemental Experimental Procedures.

### NMR Spectroscopy and Structure Calculation

Details about the NMR solution structure calculation of the Gp2-β′ jaw fragment complex are provided in the Supplemental Experimental Procedures.

### In Vitro Transcription Assays

In vitro transcription assays were conducted essentially as previously described (Cámara et al., 2010). Reactions (10 μl) were conducted using final concentrations of 100 nM Eσ^70^, 20 nM unlabeled promoter DNA probes, 0.5 mM dinucleotide primer ApA, 100 μg/ml heparin, and 3 μCi of [α-^32^P]-UTP (for *lac*UV5) or [α-^32^P]-ATP and 0.5 μM CTP (for *osmE*). Unless otherwise indicated, Gp2 and Eσ^70^ (at a 2:1 molar ratio) were always preincubated before the promoter DNA was added to the reaction. When present, R1.1 was always preincubated with Eσ^70^_ΔR1.1_ (at the indicated amounts) before addition of Gp2 and/or promoter DNA to the reaction. The reactions were resolved on a 20% (w/v) urea-denaturing polyacrylamide gel. The dried gel was visualized and quantified with the use of an FLA-5000 PhosphorImager.

### Native Gel Mobility Assays

All native mobility shift assays were conducted essentially as described previously (Cámara et al., 2010). Binding reactions (10 μl) were set up as described above.

### FRET Assays

Fluorescence emission spectra of Eσ^70^ reconstituted with σ^70^ subunit labeled at position 36 or 59 with fluorescein (σ^70∗^) were recorded with 482 excitation as previously described (Knight et al., 2005). When present, Rif and Sor (at 1 μM) were incubated with Eσ^70∗^ for 10 min at 37°C. The Eσ^70^-Gp2 complex was obtained by incubation of 1 nM Eσ^70^ and 200 nM Gp2 for 10 min at 37°C. Promoter complexes were obtained by incubation of 1 nM Eσ^70^ or Eσ^70^-Gp2 with 5 nM N25cons for 15 min at 37°C. The FRET efficiency, critical FRET radius (R_0_), and distance between fluorescein and Rif were determined as previously described (Knight et al., 2005). A previous analysis of the accuracy of similar FRET-based measurements of distances between Eσ^70^-bound Rif and fluorescein incorporated at different positions in σ^70^ indicated that the uncertainty of such a distance determination is 11%–25%, with a mean of 15% (Knight et al., 2005).

## Figures and Tables

**Table 1:** Gp2-Jaw Complex

Number of experimental restraints	1,700
Total NOE-derived	1,532
Ambiguous	476
Unambiguous	1,056
Intraresidue	468
Sequential	200
Medium-range (|i − j| ≤ 4)	73
Long-range (|i − j| > 4)	265
Intermolecular	50
TALOS, φ/ψ	168
RMSD from experimental restraints	
Distance, Å	0.023 ± 0.002
Dihedral angle, degrees	0.6 ± 0.1
RMSD from idealized covalent geometry	
Bonds, Å	0.0036 ± 0.0001
Improper angles, degrees	1.35 ± 0.06
Angles, degrees	0.54 ± 0.01
Coordinate RMSD, Å	
Backbone atoms in secondary structure	1.21 ± 0.15
Heavy atoms in secondary structure	1.47 ± 0.15
Ramachandran plot	
Residues in most favored regions, %	75.7
Residues in allowed regions, %	24.2
Residues in disallowed regions, %	0.1

RMSD, root mean-squared deviation.

## Accession Numbers

The coordinates and structure factors for the Gp2-β′ jaw fragment complex have been deposited in the Protein Data Bank (accession code 2LMC).

## Supplemental Information

Document S1. Figures S1–S5 and Supplemental Experimental Procedures
