# Supplementary material for: Structural and Mechanistic Basis for the Inhibition of Escherichia coli RNA Polymerase by T7 Gp2
Source: Mol Cell. 2012 Sep 14;47(5):755–66. doi: 10.1016/j.molcel.2012.06.013 (PMC3778932; doi:10.1016/j.molcel.2012.06.013)
Supplement: Document S1. Figures S1–S5 and Supplemental Experimental Procedures [file mmc1.pdf]

Molecular Cell, Volume *47*

Supplemental Information

Structural and Mechanistic Basis for the Inhibition of *Escherichia coli* RNA Polymerase by T7 Gp2

Ellen James, Minhao Liu, Carol Sheppard, Vladimir Mekler, Beatriz Cámara, Bing Liu, Pete Simpson, Ernesto Cota, Konstantin Severinov, Steve Matthews, and Sivaramesh Wigneshweraraj

Figure S1

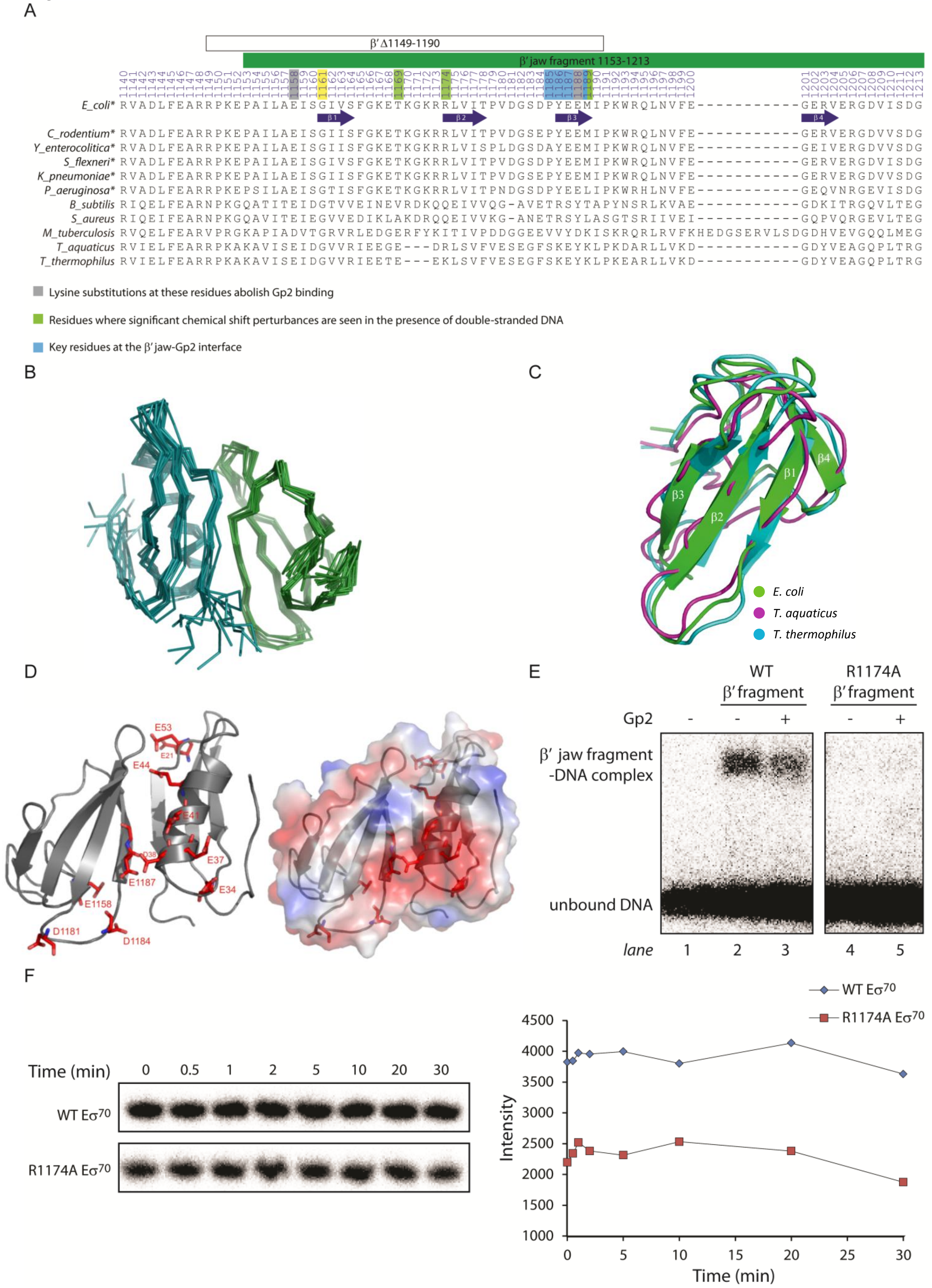

A
β′ Δ1149-1190
β′ jaw fragment 1153-1213
E_coli* RVADLFEARRPKEPAILAEISGIVSFGKETKGKRRLVITPVDGSDPYEEMIPKWRQLNVFE-----------GERVERGDVISDG
β1
β2
β3
β4
C_rodentium* RVADLFEARRPKEPAILAEISGIISFGKETKGKRRLVITPVDGSEPYEEMIPKWRQLNVFE-----------GERVERGDVVSDG
Y_enterocolitica* RVADLFEARRPKEPAILAEISGIISFGKETKGKRRLVISPLDGSDAYEEMIPKWRQLNVFE-----------GEIVERGDVVSDG
S_flexneri* RVADLFEARRPKEPAILAEISGIVSFGKETKGKRRLVITPVDGSDPYEEMIPKWRQLNVFE-----------GERVERGDVISDG
K_pneumoniae* RVADLFEARRPKEPAILAEISGIISFGKETKGKRRLVITPVDGSEPYEEMIPKWRQLNVFE-----------GERVERGDVVSDG
P_aeruginosa* RVADLFEARRPKEPSILAEISGTISFGKETKGKRRLVITPNDGSDPYEELIPKWRHLNVFE-----------GEQVNRGEVISDG
B_subtilis RIQELFEARNPKGQATITEIDGTVVEINEVRDKQQEIVVQG-AVETRSYTAPYNSRLKVAE-----------GDKITRGQVLTEG
S_aureus RIQEIFEARNPKGQAVITEIEGVVEDIKLAKDRQQEIVVKG-ANETRSYLASGTSRIIVEI-----------GQPVQRGEVLTEG
M_tuberculosis RVQELFEARVPRGKAPIADVTGRVRLEDGERFYKITIVPDDGGEEVVYDKISKRQRLRVFKHEDGSERVLSDGDHVEVGQQLMEG
T_aquaticus RVIELFEARRPKAKAVISEIDGVVRIEEGE---DRLSVFVESEGFSKEYKLPKDARLLVKD-----------GDYVEAGQPLTRG
T_thermophilus RVIELFEARRPKAKAVISEIDGVVRIEETE---EKLSVFVESEGFSKEYKLPKEARLLVKD-----------GDYVEAGQPLTRG
Lysine substitutions at these residues abolish Gp2 binding
Residues where significant chemical shift perturbances are seen in the presence of double-stranded DNA
Key residues at the β′ jaw-Gp2 interface
B
C
β1
β2
β3
β4
E. coli
T. aquaticus
T. thermophilus
D
E53
E21
E44
E41
D38
E37
E1187
E34
E1158
D1181
D1184
E
WT β′ fragment
R1174A β′ fragment
Gp2
- - + - +
β′ jaw fragment -DNA complex
unbound DNA
lane 1 2 3 4 5
F
Time (min) 0 0.5 1 2 5 10 20 30
WT Eσ70
R1174A Eσ70
WT Eσ70
R1174A Eσ70
Intensity
4500
4000
3500
3000
2500
2000
1500
1000
0 5 10 15 20 25 30
Time (min)

Figure S1, Related to Figure 1. The Structure of the Gp2-β' Jaw Fragment Complex

(A) The amino acid sequence alignment of the β' jaw domain region (*E. coli* residues 1140-1213). Bacteria marked with an asterisk are infected by T7-like phages encoding Gp2-like proteins. The region corresponding to the 1149-1190 deletion and the region of β' jaw domain (i.e. β' jaw fragment) corresponding to the solution structure determined in this study are indicated by the white and green bars, respectively, above the amino acid sequence. The localisation of the β sheets in the β' jaw fragment are indicated by the blue arrows. Highlighted in yellow is the position (G1161) at which an arginine substitution (G1161R) results in an RNAp mutant that forms RPo with significantly reduced half-life. See text and key for details on other highlighted residues.

(B) Backbone traces representing the ensemble of NMR-derived structures of the Gp2-β' jaw fragment complex (Gp2 and the β' jaw fragment is shown in cyan and green, respectively).

(C) Structural alignment of *E. coli* β' jaw fragment (residues 1153-1213; green) to the corresponding residues (1271-1328) of the β' subunit in the crystal structures of *Thermus aquaticus* (magenta; PDB code: 1HMQ) and *T. thermophilus* (turquoise; PDB code: 2BE5). Sequence identity between *E. coli* and *T. thermophilus* is 30% and the structural root-mean-square-deviation (RMSD) value for atom deviation is 1.4Å for 2BE5 (Vassylyev et al., 2005). Sequence identity between *E. coli* and *T. aquaticus* is 28% and the structural RMSD for atom deviation is 2.8Å for 1HMQ (Minakhin et al., 2001). Thus, the β' jaw domain fragment in *E. coli, T. aquaticus and T. thermophilus* are highly similar, although our NMR structure defines an extra beta strand (β4).

(D) Left: ribbons representation of the Gp2-β' jaw fragment complex in which the residues that contribute to the extended negatively charged patch are shown as sticks and labelled correspondingly (Gp2 residues = E21, E34, D37, E38, E41, E44 and E53; β' jaw fragment residues = E1158, D1181, D1184 and E1187). Right: as in (Left) but includes electrostatic surface representation of the Gp2-β' jaw fragment complex.

(E) An autoradiograph of 15% (v/v) SDS-denaturing polyacrylamide gel showing formaldehyde mediated crosslinking of the wild-type β' jaw fragment and a mutant form containing the R1174A substitution to γ^{32}P-labelled double-stranded 14bp long DNA fragment (i.e. same as the one used in Figure 2B) in the absence and presence of Gp2.

(F) Left: autoradiograph of 20% (v/v) denaturing urea-gels showing the synthesis of the ApApUpU transcript from RPo formed on the *lac*UV5 promoter by $E\sigma^{70}$ or $E\sigma^{70}$ containing the R1174A mutation in the β' (R1174A $E\sigma^{70}$) upon challenge with the DNA competitor heparin for the indicated amounts of time (minutes) Right: Graph showing the relative intensities of the bands shown in the autoradiograph (Left) plotted as a function of time.

Figure S2

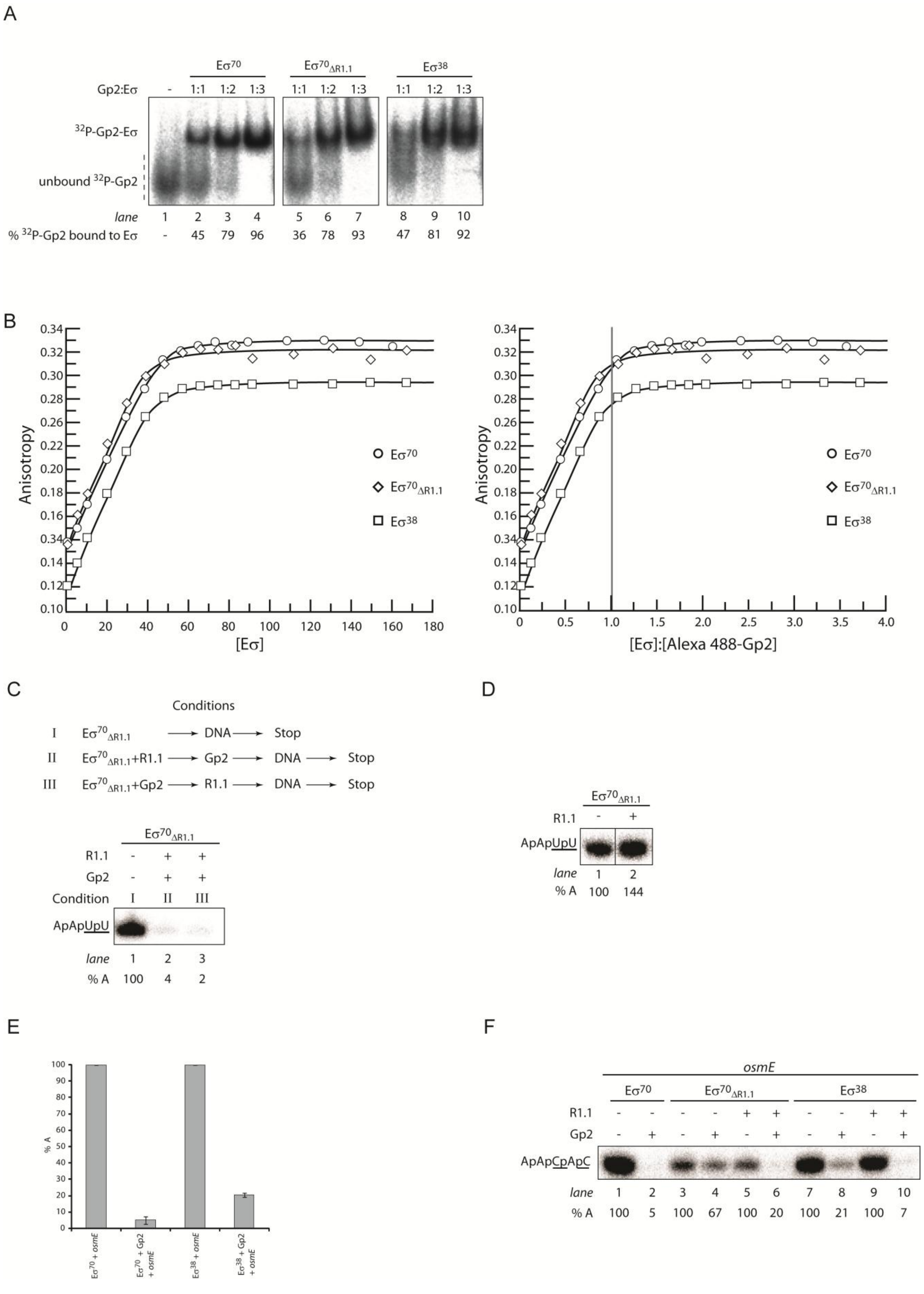

A
Eσ70
Eσ70ΔR1.1
Eσ38
Gp2:Eσ - 1:1 1:2 1:3 1:1 1:2 1:3 1:1 1:2 1:3
32P-Gp2-Eσ
unbound 32P-Gp2
lane 1 2 3 4 5 6 7 8 9 10
% 32P-Gp2 bound to Eσ - 45 79 96 36 78 93 47 81 92
B
Anisotropy
0.34 0.32 0.30 0.28 0.26 0.24 0.22 0.20 0.18 0.16 0.34 0.14 0.12 0.10
0 20 40 60 80 100 120 140 160 180
[Eσ]
Eσ70
Eσ70ΔR1.1
Eσ38
0 0.5 1.0 1.5 2.0 2.5 3.0 3.5 4.0
[Eσ]:[Alexa 488-Gp2]
C
Conditions
I Eσ70ΔR1.1 → DNA → Stop
II Eσ70ΔR1.1+R1.1 → Gp2 → DNA → Stop
III Eσ70ΔR1.1+Gp2 → R1.1 → DNA → Stop
Eσ70ΔR1.1
R1.1 - + +
Gp2 - + +
Condition I II III
ApApUpU
lane 1 2 3
% A 100 4 2
D
Eσ70ΔR1.1
R1.1 - +
ApApUpU
lane 1 2
% A 100 144
E
% A
100 90 80 70 60 50 40 30 20 10 0
Eσ70 + osmE
Eσ70 + Gp2 + osmE
Eσ38 + osmE
Eσ38 + Gp2 + osmE
F
osmE
Eσ70
Eσ70ΔR1.1
Eσ38
R1.1 - - - - + + - - + +
Gp2 - + - + - + - + - +
ApApCpApC
lane 1 2 3 4 5 6 7 8 9 10
% A 100 5 100 67 100 20 100 21 100 7

Figure S2, Related to Figure 3. Gp2 Requires R1.1 to Efficiently Inhibit RPo Formation by Eσ^{70}

(A) An autoradiograph of 4% (v/v) native polyacrylamide gel showing binding of α^{32}P-labelled Gp2 to RNAp containing σ^{70}, $\sigma^{70}_{\Delta R1.1}$ or σ^{38}. The migration positions of ^{32}P-Gp2 and ^{32}P-Gp2-Eσ complex on the native gel are indicated. Semi-quantitative analysis of the autoradiograph indicate that at equimolar amounts of ^{32}P–Gp2 and RNAp approximately equal amount of radioactivity is present in the ^{32}P-Gp2-Eσ complex indicating that Gp2 binds to RNAp containing σ^{70}, $\sigma^{70}_{\Delta R1.1}$ or σ^{38} equally well under conditions at which the *in vitro* transcription assays are conducted. The average percentage ^{32}P-Gp2 in complex with Eσ from at least five independent experiments fell within 5-8% of the value shown and is given at the bottom of the gel.

(B) Graph showing the binding of 50 nM Alexa488-fluorophore-labelled Gp2 (Alexa488-Gp2) to Eσ^{70}, E$\sigma^{70}_{\Delta R1.1}$ or Eσ^{38} shown as a function of Eσ concentration (left) and molar ratio of Alexa488-Gp2 to Eσ in the reaction (right). The fluorescence anisotropy based equilibrium binding assays were conducted as described in the supplemental experimental procedures section. In the graph on the right the vertical line indicates molar ratio of [Alexa488-Gp2]:[Eσ] at which saturation of binding occurs, thus further indicating that Gp2 binds to RNAp containing σ^{70}, $\sigma^{70}_{\Delta R1.1}$ or σ^{38} equally well.

(C) An autoradiograph of 20% (v/v) denaturing urea-gel showing the synthesis of the ApApUpU transcript (underlined nucleotides are α^{32}P labelled) from the *lac*UV5 +20/+20 promoter probe. Transcription by E$\sigma^{70}_{\Delta R1.1}$ in the absence (lanes 1) and presence (lanes 2 and 3) of R1.1 domain (at ~8 fold molar excess over $\sigma^{70}_{\Delta R1.1}$) added *in trans* either before (lane 2, condition II) or after (lane 3, condition III) Gp2 binding to the E$\sigma^{70}_{\Delta R1.1}$. The percentage of ApApUpU transcript synthesised (% A) in the reactions with the isolated R1.1 domain with respect to the reaction with no isolated R1.1 domain added is given at the bottom of the gel for each reaction.

(D) As in (C), but showing the synthesis of ApApUpU transcript by E$\sigma^{70}_{\Delta R1.1}$ in the absence (lane 1) and presence (lane 2) of the isolated R1.1 domain added *in trans*.

(E) Graph showing the amount of ApApCpApC transcript synthesised (% Transcription) from the *osmE* promoter by Eσ^{70} and Eσ^{38} in the presence of Gp2 relative to the ApApCpApC transcript synthesised its absence; the error bars show standard deviation from three independent experiments. (F) An autoradiograph of a representative 20% (v/v) denaturing gel, from which the data for (E) was obtained, showing the synthesis of the ApApCpApC transcript (underlined nucleotides are α^{32}P labelled) from the *osmE* promoter by Eσ^{70}, E$\sigma^{70}_{\Delta R1.1}$ and Eσ^{38} in the absence and presence of ~2-fold molar excess of Gp2 over RNAp. The presence of the isolated R1.1 domain *in trans* in the reactions is also indicated. The percentage of ApApCpApC transcript synthesised (% A) in the reactions with Gp2 respect to reactions where no Gp2 was added is given at the bottom of the gel for each reaction. For (C), (D) and (F) all data obtained in at least three independent experiments fell within 5% of the % A value shown.

Figure S3

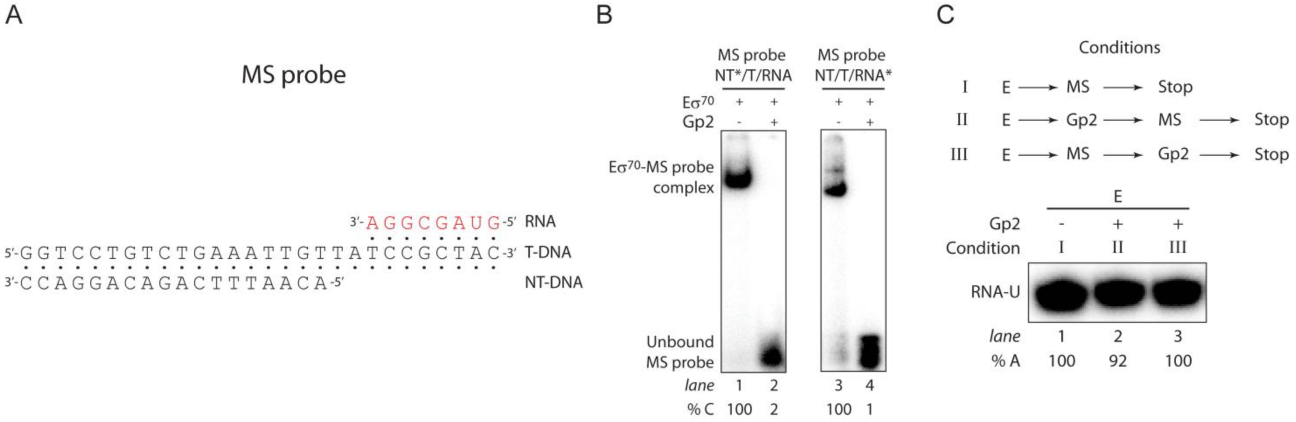

Figure S3, Related to Figure 4. Gp2 Requires R1.1 of σ^{70} but Not the Consensus Promoter DNA Sequences to Fully Inhibit RPo Formation by Eσ^{70}

(A) Nucleic acid sequence of the MS probe; shown in red is the RNA and in black the DNA components of the MS probe.

(B) An autoradiograph of 4% (v/v) native polyacrylamide gel showing binding of Eσ^{70} to the MS probe in the absence (lanes 1 and 3) and presence (lanes 2 and 4) of Gp2. In lanes 1 and 2 and lanes 3 and 4 the non-template DNA strand and the RNA template, respectively, of the MS probe is labelled with γ^{32}P (indicated by * at the top of the gel). The percentages of DNA bound by Eσ^{70} (% C) in the reactions with Gp2 with respect to reactions with no Gp2 are given at the bottom of the gel and the data obtained in at least two independent experiments fell within 3% of the % C value shown.

(C) An autoradiograph of 20% (v/v) denaturing urea-gel showing the synthesis of RNA-U from the MS probe by the core RNAp (E) in the absence (condition I) of Gp2 and when Gp2 is present either before (condition II) or after (condition III) the binding of E to the MS probe. The percentage of RNA-U synthesised (% A) in the reactions with Gp2 with respect to reactions with no Gp2 are given at the bottom of the gel for each reaction and all data obtained in at least three independent experiments fell within 5% of the % A value shown.

Figure S4

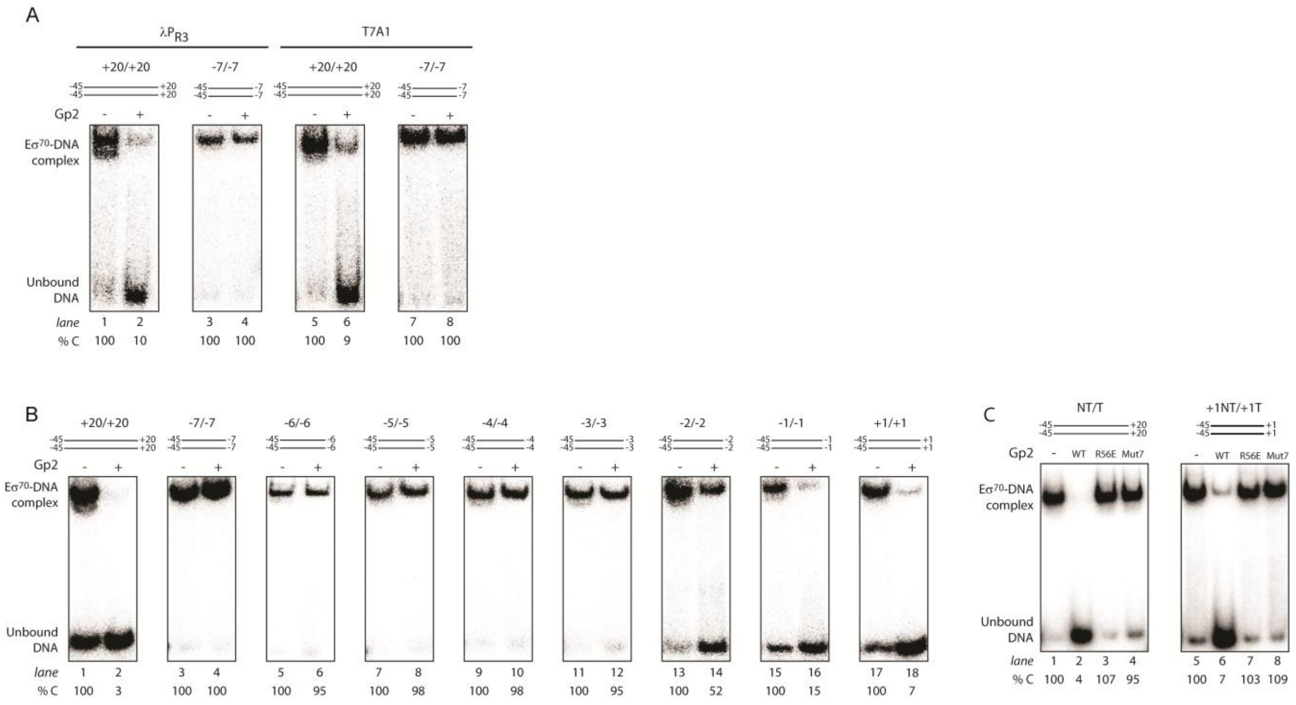

Figure S4, Related to Figure 5. Inhibition of RPo Formation by Gp2 Involves a Long-Range Antagonistic Effect on Eσ^{70}-Promoter Interactions

(A) An autoradiograph of a 4% (v/v) native polyacrylamide gel showing the binding of Eσ^{70} to the +20/+20 and -7/-7 version of the T7A1 and λPR3 promoter probes in the absence (lanes 1, 3, 5 and 7) and presence (lanes 2, 4, 6 and 8) of Gp2.

(B) An autoradiograph of 4% (v/v) native polyacrylamide gels showing the binding of Eσ^{70} in the absence (lanes 1, 3, 5, 7, 9, 11, 13, 15 and 17) and presence (lanes 2, 4, 6, 8, 10, 12, 14, 16 and 18) of Gp2 to double stranded *lac*UV5 promoter probes with different downstream end points (indicated at the top of the gel).

(C) An autoradiograph of a 4% (v/v) native polyacrylamide gel showing the binding of Eσ^{70} to the +20/+20 and +1/+1 versions of the double stranded *lac*UV5 promoter probes in the absence (lanes 1 and 5) and presence (lanes 2-4 and 6-8) of Gp2. Lanes 3 and 7 contain the RNAp binding mutant version of Gp2 ($Gp2_{R56E}$ (Cámara et al., 2010), and lanes 4 and 8 contain the functionally attenuated mutant Gp2 (Mut7 (Sheppard et al., 2011). In (A), (B) and (C), the percentages of DNA bound by

$E\sigma^{70}$ (% C) in the reactions with Gp2 with respect to reactions with no Gp2 are given at the bottom of the gel and the data obtained in at least two independent experiments fell within 3% of the % C value shown.

Figure S5

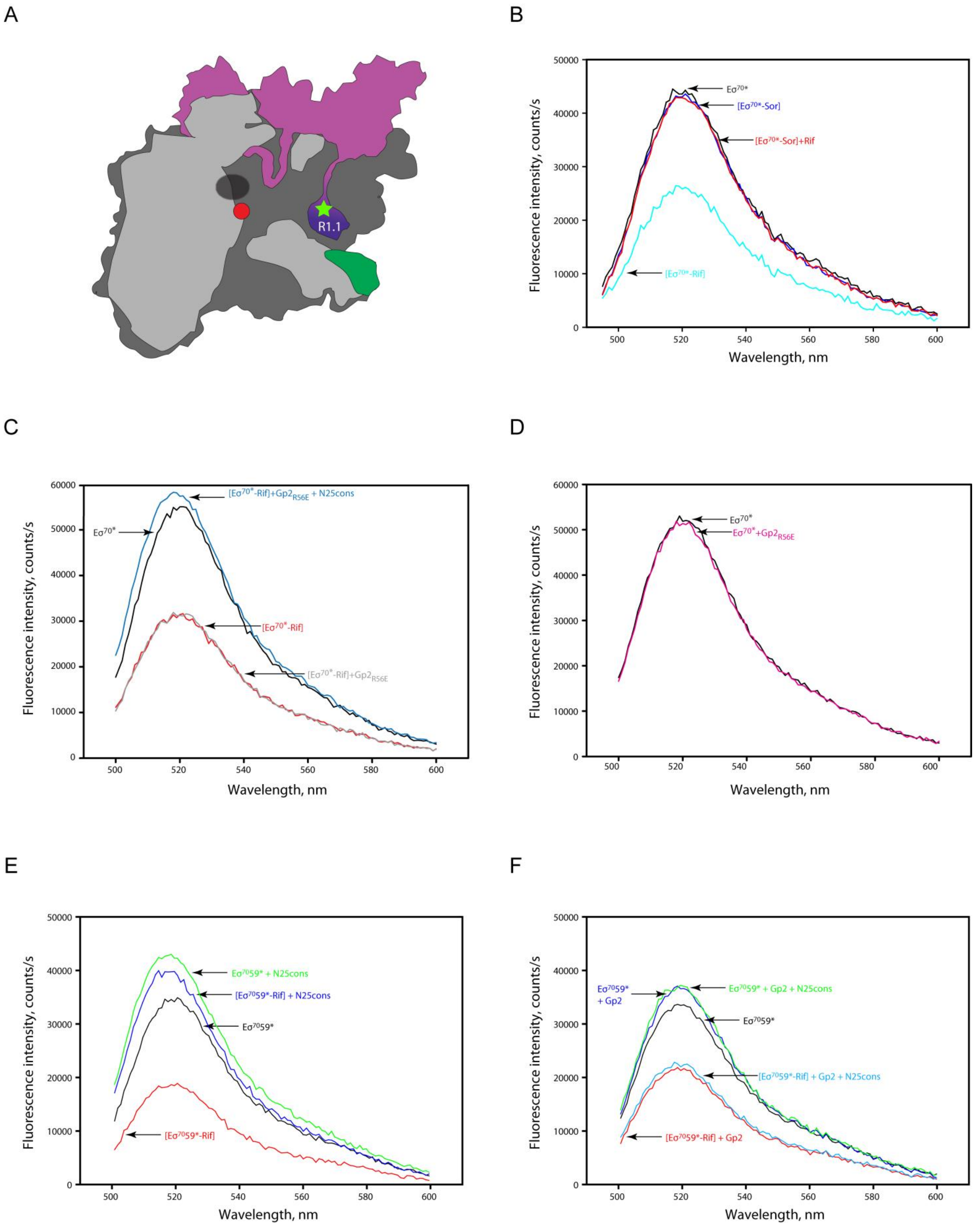
A
R1.1
B
Eσ70*
[Eσ70*-Sor]
[Eσ70*-Sor]+Rif
[Eσ70*-Rif]
Fluorescence intensity, counts/s
Wavelength, nm
C
[Eσ70*-Rif]+Gp2R56E + N25cons
Eσ70*
[Eσ70*-Rif]
[Eσ70*-Rif]+Gp2R56E
Fluorescence intensity, counts/s
Wavelength, nm
D
Eσ70*
Eσ70*+Gp2R56E
Fluorescence intensity, counts/s
Wavelength, nm
E
Eσ7059* + N25cons
[Eσ7059*-Rif] + N25cons
Eσ7059*
[Eσ7059*-Rif]
Fluorescence intensity, counts/s
Wavelength, nm
F
Eσ7059* + Gp2
Eσ7059* + Gp2 + N25cons
Eσ7059*
[Eσ7059*-Rif] + Gp2 + N25cons
[Eσ7059*-Rif] + Gp2
Fluorescence intensity, counts/s
Wavelength, nm

Figure S5, Related to Figure 6. Gp2 Appropriates R1.1 to Efficiently Inhibit RPo Formation by Eσ^{70}

(A) Cartoon showing the approximate locations of Rif (black oval) and fluorescein (green star) attached to R1.1 of σ^{70} (either at amino acid residue 36 or 59) in RNAp used in the FRET assays (RNAp colour coding as in Figure 1 and 6).

(B) Measurement of FRET between fluorescein incorporated into σ^{70} at position 36 (σ^{70}*) and Rif in Eσ^{70}* and in Eσ^{70}* preincubated with 1 μM Sorangicin A [Eσ^{70}*-Sor].

(C) Measurement of FRET between fluorescein incorporated into σ^{70} at position 36 (σ^{70}*) and Rif during RPo formation in the presence of the R56E mutant Gp2 ($Gp2_{R56E}$). $Gp2_{R56E}$ does not change the FRET efficiencies during RPo formation ((C); compare the curves with that shown in Figure 6B) or the Eσ^{70}* fluorescence spectrum ((D); compare the curves with that shown in Figure 6B).

(E) Measurement of FRET between fluorescein incorporated into σ^{70} at position 59 (σ^{70}59*) and Rif in Eσ^{70}59* and Eσ^{70}59* in complex with N25cons promoter DNA (compare with Figure 6A and 6C).

(F) As in (E) with Eσ^{70}* preincubated with 100 nM Gp2 for 10 min (compare with Figure 6B and 6C). For (B)-(F), the fluorescence emission spectra are recorded with 482 nm excitation.

SUPPLEMENTAL EXPERIMENTAL PROCEDURES

Proteins and promoter templates.

Overexpression plasmids for σ^{70} (pT7σ) (Nechaev and Severinov, 1999), $\sigma^{70}_{\Delta R1.1}$ (pQE30-T-$\sigma^{70}\Delta100$) (Wilson and Dombroski, 1997), R1.1 (pET-15b-R1.1) (Wilson and Dombroski, 1997), σ^{38} (pBAD-*rpoS*) (Bordes et al., 2000), Gp2 (pSW33-Gp2) (Cámara et al., 2010) and core RNAp subunits (pVS10) (Belogurov et al., 2007) were constructed and proteins expressed and purified by nickel-affinity chromatography exactly as previously described. The gene encoding β' jaw fragment (*E. coli* residues 1153-1213) was amplified from pVS10 and expressed from pET28b+ plasmid (Novagen) as an amino-terminally 6His-tagged protein and purified as described above. Unlabelled and $\gamma^{32}P$ end labelled promoter probes were reconstituted using commercially synthesised oligonucleotides as previously described (Wigneshweraraj et al., 2003). Fluorescein was incorporated into σ^{70} R1.1 either at amino acid position 36 or 59 as described previously (Knight et al., 2005). Sequence details of all the PCR primers and oligonucleotides used in this study are available from the authors upon request.

NMR Spectroscopy and Structure Calculation.

For NMR sample preparation eluted purified Gp2 underwent buffer exchange via dialysis into NMR buffer (10 mM Tris-HCl, 50 mM NaCl, 10 mM DTT, pH 6.5) and concentrated to 0.25-0.5 ml. NMR Spectra were processed with NMRPipe (Delaglio et al., 1995) and analysed using NMRView (Johnson and Blevins, 1994) and our in-house assignment routines (Marchant et al., 2008). A total of 18 intermolecular NOEs, providing unambiguous 3D information, were manually assigned from the ^{13}C-NOESY data. The ARIA protocol (Linge et al., 2003) was used for completion of the NOE assignment and structure calculation. Dihedral angle restraints derived from TALOS were also implemented (Shen et al., 2009). The frequency window tolerance for assigning NOEs was ±0.05 ppm and ±0.08 ppm for direct and indirect proton dimensions and ±0.5 ppm and

±1 ppm for nitrogen and carbon dimensions, respectively. The ARIA parameters, p, Tv, and Nv, were set to default values. A slow cooling step was invoked using 36000 dynamic steps of 0.003 ps (Fossi et al., 2005). The 10 lowest energy structures had no NOE violations greater than 0.5Å and dihedral angle violations greater than 5°. The structural statistics are presented in Table S1. For NMR mapping experiments, either ^{15}N or ^{15}N^{13}C-labelled Gp2 was prepared in the NMR buffer. Unlabelled double-stranded DNA or β' jaw fragment in the same buffer were introduced at several steps up to a saturating molar excess and 2D ^{1}H-^{15}N HSQC spectra were recorded at each stage under identical experimental conditions.

Native gel mobility assays with ^{32}P-Gp2

All native mobility shift assays to determine the ability of Gp2 to bind to Eσ reconstituted with σ^{70}, $\sigma^{70}_{\Delta R1.1}$ or σ^{38} were conducted essentially as described previously using ^{32}P-labelled Gp2 (Cámara et al., 2010). Binding reactions (10 μl) with 50, 100 and 150 nM Eσ (reconstituted in the presence of ~4-fold molar excess of σ) and 50 nM ^{32}P-Gp2 were setup in buffer R (see Experimental Procedures in main text), incubated for 5 minutes at 37 °C and analysed on a 4.5% (w/v) native polyacrylamide gel. The gel was run for 45–60 min at 100 V at 37 °C and then dried. ^{32}P-Gp2-Eσ^{70} complexes were visualized and quantified using a Fuji PhosphorImager.

Fluorescence anisotropy equilibrium binding assays

The equilibrium binding assays were conducted exactly as described previously (Bellamy and Baldwin, 2001; Sheppard et al., 2011). Briefly, the assays were done at 37 °C in a reaction volume of 400 μl in fluorescence reaction buffer (40 mM Tris-HCl, pH 8.0, 50 mM NaCl, 10 mM $MgCl_2$, 5% (v/v) glycerol, 0.02% (v/v) Tween 20). Small amounts of the Eσ (reconstituted *in situ* with ~4-fold molar excess of σ over E) were titrated into 50 nM Alexa 488-fluorophorelabelled Gp2. Five measurements of anisotropy were averaged and each protein titration was done in at least duplicate. Data were fit using the tight binding equation in the Grafit 5 software (Erithacus Software) as

described (Bellamy and Baldwin, 2001). Figures S2B and S2C show the increase in anisotropy of Gp2 as a function of the concentration of Eσ (Figure S2B) or ratio of Eσ:Gp2 (Figure S2C). In S2C the Eσ:Gp2 ratio at which saturation occurs is indicated by the dashed line.

Formaldehyde crosslinking assays

The assay shown in Figure S1E was conducted in a total reaction volume of 10 µl, in HGNED buffer (25 mM HEPES, pH 8.0, 1 mM DTT, 100 mM NaCl, 0.2 mM EDTA, 0.05% (v/v) NP-40 and 10% glycerol) containing, where present, 2.5 µM β' jaw fragment (either WT or R1174A mutant) and 2.5 µM Gp2. A 14bp ^{32}P-labelled double-stranded DNA probe (exactly as the one used for the experiments shown in Figure 3B and 3C) was added to the β' jaw fragment or to premixed β' jaw fragment-Gp2 complex and left for 5 minutes at 37 °C before the addition of 60 µM formaldehyde (Sigma). The reaction was left for 1 minute before the addition of 2 µl stop solution (1M Tris-Cl, pH 7.5 and 1M glycine). Reactions were run on a 15% (w/v) SDS-denaturing gel and the β' jaw fragment-DNA complexes were visualised using a Fuji PhosphorImager.

SUPPLEMENTAL REFERENCES

Bellamy, S.R., and Baldwin, G.S. (2001). A kinetic analysis of substrate recognition by uracil-DNA glycosylase from herpes simplex virus type 1. Nucleic Acids Res *29*, 3857-3863.

Belogurov, G.A., Vassylyeva, M.N., Svetlov, V., Klyuyev, S., Grishin, N.V., Vassylyev, D.G., and Artsimovitch, I. (2007). Structural basis for converting a general transcription factor into an operon-specific virulence regulator. Mol Cell *26*, 117-129.

Bordes, P., Repoila, F., Kolb, A., and Gutierrez, C. (2000). Involvement of differential efficiency of transcription by esigmas and esigma70 RNA polymerase holoenzymes in growth phase regulation of the Escherichia coli osmE promoter. Molecular microbiology *35*, 845-853.

Cámara, B., Liu, M., Reynolds, J., Shadrin, A., Liu, B., Kwok, K., Simpson, P., Weinzierl, R., Severinov, K., Cota, E., *et al.* (2010). T7 phage protein Gp2 inhibits the Escherichia coli RNA polymerase by antagonizing stable DNA strand separation near the transcription start site. Proc Natl Acad Sci U S A *107*, 2247-2252.

Knight, J.L., Mekler, V., Mukhopadhyay, J., Ebright, R.H., and Levy, R.M. (2005). Distance-restrained docking of rifampicin and rifamycin SV to RNA polymerase using systematic FRET measurements: developing benchmarks of model quality and reliability. Biophysical journal *88*, 925-938.

Minakhin, L., Bhagat, S., Brunning, A., Campbell, E.A., Darst, S.A., Ebright, R.H., and Severinov, K. (2001). Bacterial RNA polymerase subunit omega and eukaryotic RNA polymerase subunit RPB6 are sequence, structural, and functional homologs and promote RNA polymerase assembly. Proceedings of the National Academy of Sciences of the United States of America *98*, 892-897.

Nechaev, S., and Severinov, K. (1999). Inhibition of Escherichia coli RNA polymerase by bacteriophage T7 gene 2 protein. Journal of molecular biology *289*, 815-826.

Sheppard, C., Camara, B., Shadrin, A., Akulenko, N., Liu, M., Baldwin, G., Severinov, K., Cota, E., Matthews, S., and Wigneshweraraj, S.R. (2011). Inhibition of Escherichia coli RNAp by T7 Gp2 protein: role of negatively charged strip of amino acid residues in Gp2. Journal of molecular biology *407*, 623-632.

Vassylyev, D.G., Svetlov, V., Vassylyeva, M.N., Perederina, A., Igarashi, N., Matsugaki, N., Wakatsuki, S., and Artsimovitch, I. (2005). Structural basis for transcription inhibition by tagetitoxin. Nature structural & molecular biology *12*, 1086-1093.

Wigneshweraraj, S.R., Nechaev, S., Bordes, P., Jones, S., Cannon, W., Severinov, K., and Buck, M. (2003). Enhancer-dependent transcription by bacterial RNA polymerase: the beta subunit downstream lobe is used by sigma 54 during open promoter complex formation. Methods in enzymology *370*, 646-657.

Wilson, C., and Dombroski, A.J. (1997). Region 1 of sigma70 is required for efficient isomerization and initiation of transcription by Escherichia coli RNA polymerase. Journal of molecular biology *267*, 60-74.
